# Genetic Polymorphisms in Endothelin-1 as Predictors for Long-Term Survival and the Cardiac Index in Patients Undergoing On-Pump Cardiac Surgery

**DOI:** 10.1371/journal.pone.0131155

**Published:** 2015-06-29

**Authors:** Ashham Mansur, Maximilian Steinau, Aron Frederik Popov, Sinisa Milenovic, Christian Bireta, Alexander Weymann, Hanna Schotola, Christoph H. Wiese, Tim Beissbarth, Mladen Tzvetkov, José Hinz

**Affiliations:** 1 Department of Anesthesiology, University Medical Center, Georg August University, Robert-Koch-Str.40, D-37075 Goettingen, Germany; 2 Department of Cardiothoracic Transplantation & Mechanical Support, Royal Brompton and Harefield Hospital, Harefield, Hill End Road, UB9 6JH London, United Kingdom; 3 Department of Thoracic Cardiovascular Surgery, University Medical Center, Georg August University, Robert-Koch-Str.40, D-37075 Goettingen, Germany; 4 Department of Anesthesiology, University of Regensburg, Franz-Josef-Strauß-Allee 11, D-93053 Regensburg, Germany; 5 Department of Medical Statistics, University Medical Center, Georg August University, Robert-Koch-Str.40, D-37075 Goettingen, Germany; 6 Institute of Clinical Pharmacology, University Medical Center, Georg August University, Robert-Koch-Str.40, D-37075 Goettingen, Germany; Institute for Clinical Epidemiology and Applied Biometry, GERMANY

## Abstract

Genetic variants within the endothelin-1 gene (*EDN1*) have been associated with several cardiovascular diseases and may act as genetic prognostic markers. Here, we explored the overall relevance of *EDN1* polymorphisms for long-term survival in patients undergoing on-pump cardiac surgery. A prospectively collected cohort of 455 Caucasian patients who underwent cardiac surgery with cardiopulmonary bypass was followed up for 5 years. The obtained genotypes and inferred haplotypes were analyzed for their associations with the five-year mortality rate (primary endpoint). The *EDN1* T-1370G and K198N genotype distributions did not deviate from Hardy–Weinberg equilibrium and the major allele frequencies were 83% and 77%, respectively. The cardiovascular risk factors were equally distributed in terms of the different genotypes and haplotypes associated with the two polymorphisms. The five-year mortality rate did not differ among the different *EDN1* T-1370G and K198N genotypes and haplotypes. Haplotype analysis revealed that carriers of the G-T (compound *EDN1* T-1370G G/K198N T) haplotype had a higher cardiac index than did non-carriers (p = 0.0008); however, this difference did not reach significance after adjusting for multiple testing. The results indicate that common variations in *EDN1* do not act as prognostic markers for long-term survival in patients undergoing on-pump cardiac surgery.

## Introduction

Endothelin (ET) is a strongly vasoconstrictive peptide produced by vascular endothelial cells[1]. The three isoforms of ET (ET-1, ET-2, and ET-3) are encoded by three separate genes[2] and are produced by endothelial cells and other tissues[3]. The ET-1 gene (*EDN1*) is localized on chromosome 6, spans 5.5 kb in length, and contains five exons and four introns. *EDN1* has been identified as a strong candidate for involvement in cardiovascular diseases, including essential hypertension. Given its vasoconstrictive effects on vessels and hypertrophic impacts on myocardial tissue, the expression of ET-1 has been considered an important risk factor for hypertension[4, 5]. Moreover, ET-1 is assumed to promote vascular cell growth in an autocrine and paracrine manner through two receptor subtypes[6]. Earlier studies have revealed that individuals with arterial hypertension express significantly higher ET-1 levels than do normotensive individuals[7, 8]. Furthermore, Minami et al. demonstrated associations of carotid atherosclerosis and asymptomatic cerebrovascular lesions with elevated ET-1 levels in patients with arterial hypertension[9]. Additionally, ET-1 was shown to induce hypertrophy in vitro by increasing neonatal rat ventricular cardiomyocyte cell volumes and cellular protein synthesis [10]. Additionally, an association between the plasma ET-1 concentration and the severity of left ventricular hypertrophy (LVH) was demonstrated in humans[10]. Based on these findings, ET-1 is thought to play an important role in the etiologies of hypertension, atherosclerosis, and cardiovascular disease as well as other vascular events (VEs). A common polymorphism of *EDN1* is a G-to-T substitution that causes a lysine to asparagine change at codon 198 (K198N); in large populations, this polymorphism was found to interact with the body mass index (BMI) in association with blood pressure[11–13]. According to these earlier studies, T-allele carriers exhibit the greatest increases in blood pressure. Furthermore, Iglarz et al. confirmed the impact of the K198N polymorphism on vascular reactivity in humans and found that a sub-threshold ET-1 concentration potentiated phenylephrine-induced vasoconstriction to a significantly higher degree in T-allele carriers[14]. Another common polymorphism, *EDN1* T-1370G, is located in the 5' flanking promoter region of the gene and may therefore be involved in its differential transcriptional regulation[15]. Carriers of the *EDN1* T-1370G G allele presented with significantly increased left ventricular hypertrophy[16]. This polymorphism has also been associated with bronchial hyperreactivity and asthma in two independent Caucasian cohorts[17].

Given this background, the potential clinical and functional relevance of *EDN1* T-1370G and K198N in Caucasians, these two polymorphisms were selected for an assessment of their clinical impacts on a cohort of Caucasian patients undergoing on-pump cardiac surgery.

This study aimed to explore whether *EDN1* T-1370G and K198N or their haplotype combinations might impact the long-term (5-year) survival (primary outcome) of patients following cardiac surgery with cardiopulmonary bypass.

The results indicate that common variations in EDN1 do not act as prognostic markers for long-term survival in patients undergoing on-pump cardiac surgery.

## Patients and Methods

### Patients

This study included adult Caucasian patients who were admitted to the University Medical Center Goettingen (UMG) and underwent cardiac surgery with cardiopulmonary bypass from 2006–2007. Patients with known neoplasms were excluded. This study conformed with the ethical principles of the Declaration of Helsinki (Seoul, 2008) and was approved by the University of Goettingen ethics committee in Goettingen, Germany. Written informed consent was obtained either from the patients or their legal representatives.

### Data collection

After enrollment, the patients were followed up for 5 years and their deaths were recorded as the primary outcome variable. The cardiac index (CI), systemic (SVRI), and pulmonary vascular resistance (PVRI) indices were calculated from standard formulas as secondary outcome parameters. An intraaortic balloon pump (IABP) and extracorporeal membrane oxygenation (ECMO) requirement were also assessed as secondary endpoints. Similarly, the length of the hospital and intensive care unit (ICU) stay and the in-hospital mortality were also recorded as secondary parameters.

Additionally, the postoperative course, including the complete intensive care unit stay, was assessed for relevant pulmonary and hemodynamic parameters. These parameters comprised the oxygenation index (P_a_O_2_/F_i_O_2_), positive end-expiratory pressure (PEEP), partial pressure of carbon dioxide (PCO_2_), arterial pH, lung compliance, pulmonary infiltrates, intraoperative cross-clamp time, cardiopulmonary bypass time, lung injury score, Acute Physiology and Chronic Health Evaluation Score (APACHE II), and Simplified Acute Physiology Score (SAPS II). The hemodynamic measurements included the heart rate (HR), mean arterial pressure (MAP), central venous pressure (CVP), mean pulmonary artery pressure (PAP), and pulmonary capillary wedge pressure (PCWP). Moreover, catecholamine support and amiodarone, cortisone, nitroglycerin, or vasopressin administrations were recorded. The use of blood cell suspensions and blood products was also recorded over the observation period.

### Genotyping

Using EDTA-treated whole blood that was collected before surgery, genomic DNA was extracted from the peripheral blood lymphocytes using a commercially available kit, as described previously[18]. The *EDN1* T-1370G (rs1800541) and K198N (rs5370) polymorphisms were genotyped using pre-designed TaqMan genotyping assays according to the manufacturer’s instructions (assay IDs C___7464900_10 and C____598677_1_, respectively; Life Technologies, Darmstadt, Germany). Twenty percent of the samples were genotyped in duplicate, and complete concordance of the genotyping results was achieved. The DNA sample identities were controlled by sex typing[19], and samples exhibiting sex-mismatches (8 out of 463) were excluded from the analyses.

EDN1 haplotypes were inferred using the Bayesian statistical-based program PHASE version 2.1[20, 21]. *EDN1* haplotype estimation was performed with ten independent runs using the seeds 2, 1536, 2936, 3123, 4957, 5283, 6757, 7992, 8633, and 9045. The most likely combinations of individual pairs of haplotypes (best pairs) were used in the further analyses. The minimal probability for the most likely combinations used was 99.5%.

### Statistical analysis

The statistical analysis was performed using Statistica (StatSoft, Tulsa, Oklahoma, USA, version 10) or R software (The R Foundation for Statistical Computing, version 3.0.0). Continuous variables are presented as the mean ± standard deviation, and categorical variables are presented as an absolute number or percentage. Continuous variables were compared using the Kruskal–Wallis test and the F-test. Significance based on contingency tables was calculated using a two-sided Fisher’s exact test or the chi-square test as appropriate. Allele frequencies in the study population were counted and compared to an expected distribution in a normal population according to Hardy–Weinberg equilibrium and were analyzed using the chi-square test. The time-to-event data were compared using the log–rank test from the R survival package. To adjust for possible effects of confounders we used a multivariate Cox regression model with the additional confounder variables Age, SAPS II, Euroscore, APACHE II; the p-value for a significant genotype effect was assessed then using ANOVA. Analysis was performed using the R package survival. A p-value <0.05 was considered a statistically significant difference. The significance levels were adjusted according to the Bonferroni correction for multiple hypothesis testing. A complete list of all tests that were performed and the corresponding test results are provided as S2 Tables.

## Results

### Baseline characteristics

A total of 455 adult Caucasian patients who underwent cardiac surgery with CPB were enrolled in this study. The *EDN1* T-1370G and K198N polymorphisms were successfully genotyped in all subjects. The genotype distributions of *EDN1* T-1370G and K198N were 311:130:14 (TT:TG:GG) and 267:170:18 (GG:GT:TT), respectively, which is consistent with Hardy–Weinberg equilibrium (p = 0.9957 and p = 0.3632, respectively). The observed minor allele frequencies of 17.4% (T-1370G) and 22.2% (K198N) were very close to those reported for reference populations like CEU from the HapMap project (17.5% and 24.5%, respectively; dbSNP database, accession numbers ss66862052 and ss68965564, respectively). The *EDN1* haplotype analysis revealed four common haplotypes (*1, *2, *3, and *4; Table 1). The baseline patient characteristics included age, gender, BMI, smoking habits, hypertension, history of diabetes, renal disorder, hypercholesterolemia, positive family history of cardiovascular disorders, left ventricular ejection fraction, peripheral disease, history of neurocerebral events, pulmonary hypertension, and chronic obstructive pulmonary disease. Additionally, the use of preoperative medications, the urgency for surgery, associated cardiac surgical procedures, and additive Euroscores were recorded. There were almost no significant differences in the baseline characteristics S1 Tables.

**Table 1:** *EDN1* haplotype distribution.

Haplotype	T-1370G/K198N	Number of haplotypes	n(%)
H1	T-G	0	21(4.6)
		1	172(37.8)
		2	262(57.6)
H2	G-T	0	316(65.6)
		1	128(28.1)
		2	11(2.4)
H3	T-T	0	399(87.7)
		1	56(12.3)
		2	0(0.0)
H4	G-G	0	447(98.2)
		1	8(1.8)
		2	0(0.0)

### Long-term mortality

**Fig 1:**
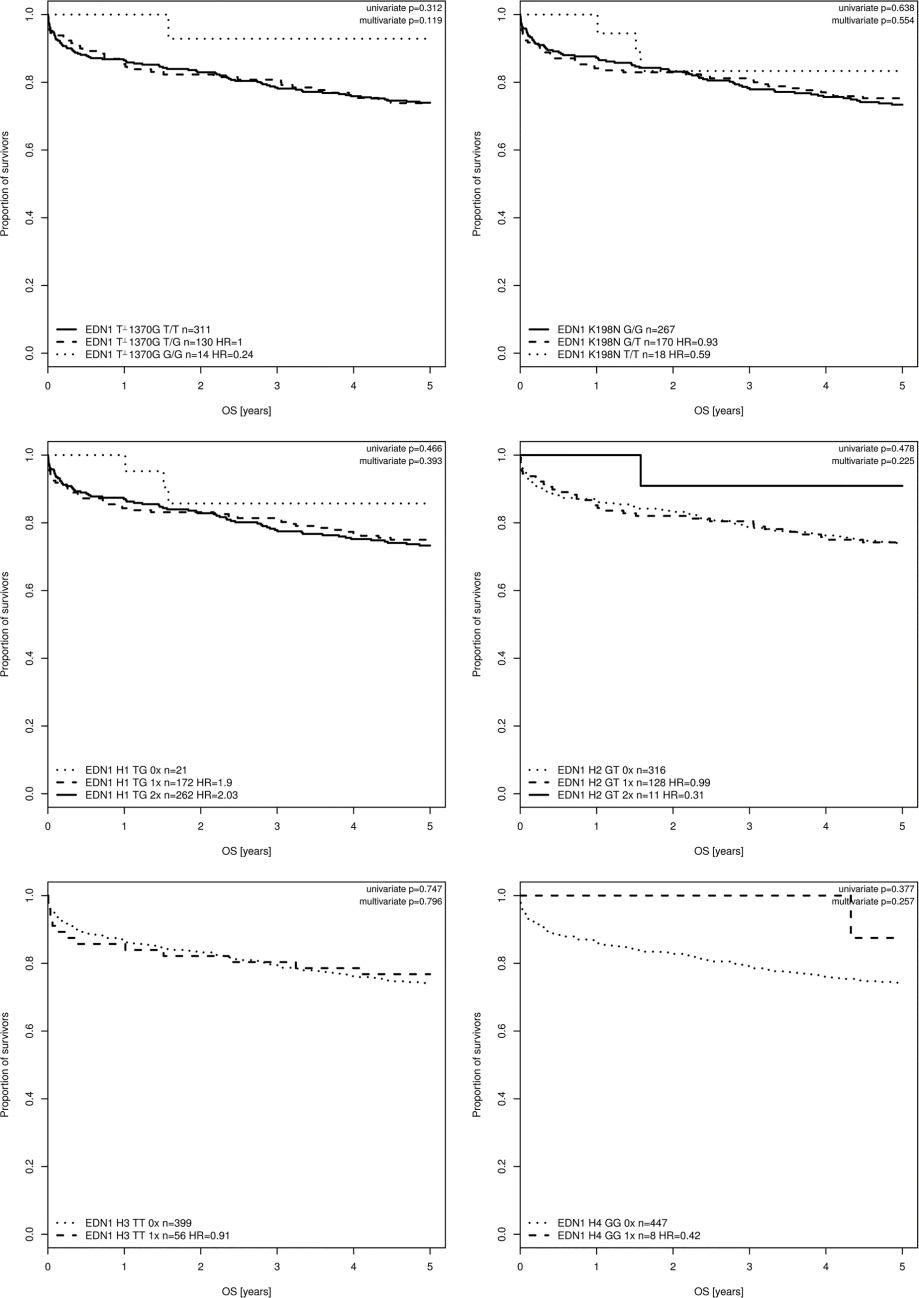
Kaplan–Meier survival analysis. The Kaplan–Meier curves demonstrating survival were censored at day 90 for the *EDN1* T-1370G and *EDN1* K198N genotypes and haplotypes. The risk of mortality did not differ between the different patient groups in this study.

In all cases (100%), long-term follow-up (5 years) data were available for the initial survivors. The overall mortality rate was 25.5% (n = 116), and there were no significant differences in the mortality rate with respect to the genotype or haplotype distributions (Table 2, Fig 1). We performed an additional multivariate Cox regression analysis with potential confounders that have and influence on mortality (i.e. Age, SAPS II, Euroscore, APACHE II). The Euroscore (European System for Cardiac Operative Risk Evaluation) is a specific risk model which allows a reliable assessment of the risk of death after cardiac surgery[22]. SAPS II and APACHE II are widely used standard morbidity scores in critically ill patients[23]. Multivariate Cox regression analysis revealed no significant results (Fig 1).

**Table 2:** Long-term mortality with respect to the *EDN1* genotypes and haplotypes.

	Mortality(%)	p-value
EDN1(T-1370G)		0.312
TT	26.1	
TG	26.2	
GG	7.1	
EDN1(K198N)		0.638
GG	26.6	
GT	24.7	
TT	16.7	
H1(T-G)		0.466
0	14.3	
1	25.0	
2	26.7	
H2(G-T)		0.478
0	26.0	
1	25.8	
2	9.1	
H3(T-T)		0.747
0	25.8	
1	23.2	
2	0.0	
H4(G-G)		0.377
0	25.7	
1	12.5	
2	0.0	

### Postoperative course

**Fig 2:**
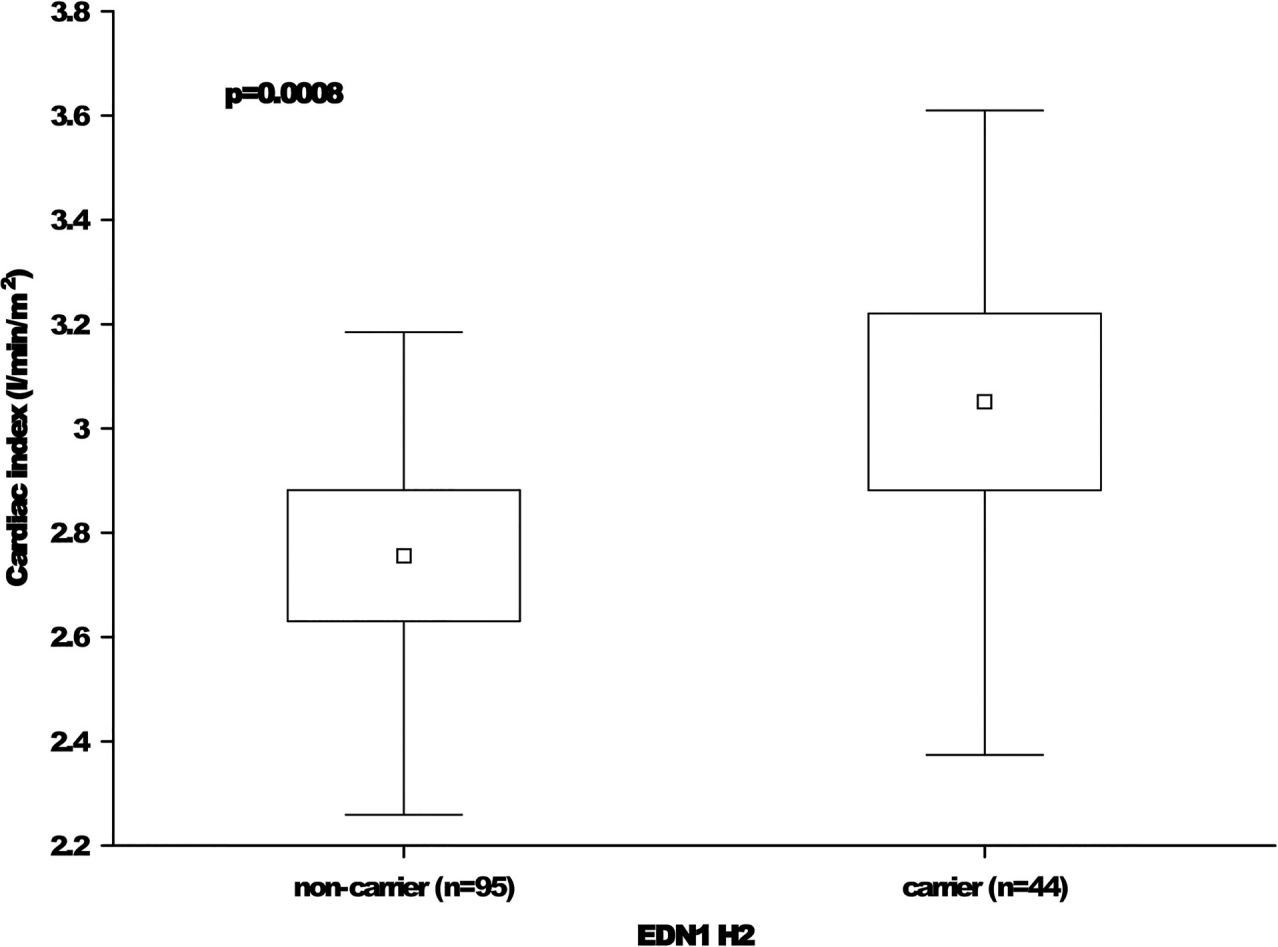
Cardiac indices with respect to the *EDN1* H2 haplotype. The means are indicated by small squares. The boxes indicate the 25th and 75th-percentile limits. The whiskers represent the minimum and maximum values. The difference between the groups is significant, as indicated by the p-value.

An exploratory analysis of the secondary endpoints revealed some statistical trends in several clinical parameters with respect to the genotype and haplotype distributions S2 Tables. The most interesting result was the increased cardiac index observed among *EDN1* H2 carriers relative to *EDN1* H2 non-carriers (3.0±0.5 and 2.7±0.6, respectively; p = 0.0008; Fig 2). Furthermore, *EDN1* K198N TT patients had higher urine output levels than did GG and GT patients (1.2±0.4, 0.9±0.5, and 1.0±0.5, respectively; p = 0.0160). However, these differences did not reach significance after adjusting for multiple testing.

### In-hospital mortality

The mean postoperative ICU and hospital length of stay was also comparable among the genotype carriers. The overall in-hospital mortality rate was 7.9% for all patients included in this study (36 of 455) and did not reach statistical significance when compared with respect to the genotypes and haplotypes. Furthermore, the in-hospital mortality rate remained uniform across urgent and emergency cases, and no significant correlation was observed among the genotypes or haplotypes S2 Tables.

## Discussion

This study examined the associations of the *EDN1* T-1370G and K198N polymorphisms and haplotypes with the long-term mortality rates (5 years) and postoperative clinical courses of patients who underwent cardiac surgery with CPB. The primary endpoint of five-year mortality did not differ among the *EDN1* T-1370G and *EDN1* K198N genotypes and haplotypes. Furthermore, no significant genotype-based differences were observed in terms of the postoperative in-hospital mortality rate. Of the secondary endpoints, carriers of the *EDN1* haplotype H2 (T-1370G G combined with K198N T) were found to have a statistical trend toward an increased cardiac index when compared with the non-carriers.

The *EDN1* T-1370G and K198N genotype distributions among the patients were similar to the database entries for healthy Caucasians and followed Hardy–Weinberg equilibrium. Neither of these genotypes nor the haplotypes was associated with any of the recorded baseline characteristics S1 Tables.

In the present study, the complete perioperative course was recorded to explore the clinical impacts of *EDN1* T-1370G and K198N on the secondary outcome parameter of perioperative and postoperative morbidity. Morbidity was assessed using several relevant clinical measurements with an emphasis on the pulmonary and hemodynamic parameters and the requirement of catecholamine and cardiac organ support S2 Tables.

The most interesting finding was the increased cardiac index values observed in *EDN1* H2 carriers when compared the values of *EDN1* H2 non-carriers (3.0±0.5 and 2.7±0.6, respectively; p = 0.0008; Fig 2). This association provides the first indication for a clinical impact of the *EDN1* G-T haplotype on the cardiac indices of patients undergoing on-pump cardiopulmonary bypass procedures. This observation should be verified in other cohorts to validate its clinical relevance. Currently, we cannot explain any causality between the G-T haplotype and an increased cardiac index. Furthermore, this result must be interpreted carefully because the cardiac index is related to several factors such as catecholamine therapy use and cardiac volumetric parameters.

The observed statistical trend toward a difference in the urine outputs of the homozygous *EDN1* K198N T carriers and G carriers S2 Tables should also be further validated in independent studies. Currently, we cannot explain how the homozygous K198N TT genotype might impact the urine output. Furthermore, this polymorphism may be linked with other causal genetic variants.

During the long-term follow-up, which was 100% complete, we observed no influence of genetic variations in ET-1 on mortality. We observed an overall mortality rate of 25.5% after five years, which is typical for heterogeneous elderly cardiac surgical patients and includes cases involving emergency and combined procedures. Of particular note are observations made by Frey et al. in which G-protein alpha subunit Gene haplotypes were found to independently associate with an increased risk of death after primary coronary artery bypass graft surgery[24].

The overall in-hospital mortality rates observed in the present study were acceptable and comparable to a reported 30-day mortality rate among cardiac surgery patients[25].

When interpreting any genetic association study, several epidemiological limitations potentially leading to false-positive or false-negative findings should be considered, including an inadequate sample size or control group selection, multiple testing, and population stratification. With regard to these concerns, the strengths of our study include a relatively large population of cardiac surgery patients and a prospective cohort design that reduced the selection bias inherent in case-control studies[26].

To the best of our knowledge, this is the first study to evaluate the clinical impacts of common *EDN1* genetic variants on the long-term survival of patients undergoing cardiac surgery. According to our investigation, it might not be useful to further evaluate the clinical impacts of *EDN1* genetic variants on long term-survival in this patient group.

## Conclusions

In conclusion, the results of the present study suggest that EDN1 genetic variants do not impact the long-term survival of patients undergoing on-pump cardiac surgery. However, the observed relationship between the EDN1 haplotype 2 and the cardiac index should be verified in independent cohorts.

## Supporting Information

S1 Tables Baseline characteristics. COPD: chronic obstructive pulmonary disease; ACE: angiotensin-converting enzyme; CABG: coronary artery bypass grafting.
(DOCX)

S2 Tables Peri- and postoperative course. PaO2/FiO2: oxygenation index; PEEP: positive end-expiratory pressure; PCO2: partial pressure of carbon dioxide; APACHE II Score: Acute Physiology and Chronic Health Evaluation Score; SAPS II Score: Simplified Acute Physiology Score; HR: Heart rate; MAP: mean arterial pressure; CVP: central venous pressure; PCWP: pulmonary capillary wedge pressure; PAP: mean pulmonary artery pressure; CI: cardiac index; SVRI: systemic vascular resistance; PVRI: pulmonary vascular resistance; NTG: nitroglycerin; IABP: intraaortic balloon pump; ECMO: extracorporeal membrane oxygenation; ICU: intensive care unit.
(DOCX)

## References

[1] Yanagisawa M, Kurihara H, Kimura S, Tomobe Y, Kobayashi M, Mitsui Y, et al A novel potent vasoconstrictor peptide produced by vascular endothelial cells. Nature. 1988;332(6163):411–5. Epub 1988/03/31. 10.1038/332411a0 . 2451132

[2] Inoue A, Yanagisawa M, Kimura S, Kasuya Y, Miyauchi T, Goto K, et al The human endothelin family: three structurally and pharmacologically distinct isopeptides predicted by three separate genes. Proceedings of the National Academy of Sciences of the United States of America. 1989;86(8):2863–7. Epub 1989/04/01. 2649896 10.1073/pnas.86.8.2863 PMC287019

[3] Levin ER. Endothelins. The New England journal of medicine. 1995;333(6):356–63. Epub 1995/08/10. 10.1056/nejm199508103330607 . 7609754

[4] Schiffrin EL, Thibault G. Plasma endothelin in human essential hypertension. American journal of hypertension. 1991;4(4 Pt 1):303–8. Epub 1991/04/01. . 2059394 10.1093/ajh/4.4.303

[5] Luscher TF, Yang ZH, Diederich D, Buhler FR. Endothelium-derived vasoactive substances: potential role in hypertension, atherosclerosis, and vascular occlusion. Journal of cardiovascular pharmacology. 1989;14 Suppl 6:S63–9. Epub 1989/01/01. . 2478827

[6] Tanaka C, Kamide K, Takiuchi S, Kawano Y, Miyata T. Evaluation of the Lys198Asn and -134delA genetic polymorphisms of the endothelin-1 gene. Hypertension research: official journal of the Japanese Society of Hypertension. 2004;27(5):367–71. Epub 2004/06/17. . 15198485 10.1291/hypres.27.367

[7] Kohno M, Yasunari K, Murakawa K, Yokokawa K, Horio T, Fukui T, et al Plasma immunoreactive endothelin in essential hypertension. The American journal of medicine. 1990;88(6):614–8. Epub 1990/06/01. . 2189304 10.1016/0002-9343(90)90527-k

[8] Vanhoutte PM. Is endothelin involved in the pathogenesis of hypertension? Hypertension. 1993;21(6 Pt 1):747–51. Epub 1993/06/01. . 8500854 10.1161/01.hyp.21.6.747

[9] Minami S, Yamano S, Yamamoto Y, Sasaki R, Nakashima T, Takaoka M, et al Associations of plasma endothelin concentration with carotid atherosclerosis and asymptomatic cerebrovascular lesions in patients with essential hypertension. Hypertension research: official journal of the Japanese Society of Hypertension. 2001;24(6):663–70. Epub 2002/01/05. . 11768725 10.1291/hypres.24.663

[10] Cullen JP, Bell D, Kelso EJ, McDermott BJ. Use of A-192621 to provide evidence for involvement of endothelin ET(B)-receptors in endothelin-1-mediated cardiomyocyte hypertrophy. European journal of pharmacology. 2001;417(3):157–68. Epub 2001/05/04. . 11334846 10.1016/s0014-2999(01)00905-0

[11] Tiret L, Poirier O, Hallet V, McDonagh TA, Morrison C, McMurray JJ, et al The Lys198Asn polymorphism in the endothelin-1 gene is associated with blood pressure in overweight people. Hypertension. 1999;33(5):1169–74. Epub 1999/05/20. . 10334806 10.1161/01.hyp.33.5.1169

[12] Asai T, Ohkubo T, Katsuya T, Higaki J, Fu Y, Fukuda M, et al Endothelin-1 gene variant associates with blood pressure in obese Japanese subjects: the Ohasama Study. Hypertension. 2001;38(6):1321–4. Epub 2001/12/26. . 11751711 10.1161/hy1101.095333

[13] Jin JJ, Nakura J, Wu Z, Yamamoto M, Abe M, Tabara Y, et al Association of endothelin-1 gene variant with hypertension. Hypertension. 2003;41(1):163–7. Epub 2003/01/04. . 12511547 10.1161/01.hyp.0000043680.75107.cf

[14] Iglarz M, Benessiano J, Philip I, Vuillaumier-Barrot S, Lasocki S, Hvass U, et al Preproendothelin-1 gene polymorphism is related to a change in vascular reactivity in the human mammary artery in vitro. Hypertension. 2002;39(2):209–13. Epub 2002/02/16. . 11847185 10.1161/hy0202.103442

[15] Pinto-Sietsma SJ, Herrmann SM, Schmidt-Petersen K, Niu T, Hillege HL, Janssen WM, et al Role of the endothelin-1 gene locus for renal impairment in the general nondiabetic population. Journal of the American Society of Nephrology: JASN. 2003;14(10):2596–602. Epub 2003/09/30. . 14514737 10.1097/01.asn.0000089827.03201.8e

[16] Dong Y, Wang X, Zhu H, Treiber FA, Snieder H. Endothelin-1 gene and progression of blood pressure and left ventricular mass: longitudinal findings in youth. Hypertension. 2004;44(6):884–90. Epub 2004/10/27. 10.1161/01.HYP.0000147824.08621.a6 . 15505112

[17] Zhu G, Carlsen K, Carlsen KH, Lenney W, Silverman M, Whyte MK, et al Polymorphisms in the endothelin-1 (EDN1) are associated with asthma in two populations. Genes and immunity. 2008;9(1):23–9. Epub 2007/10/26. 10.1038/sj.gene.6364441 . 17960156

[18] Liakopoulos OJ, Dorge H, Popov AF, Schmitto JD, Cattaruzza M, Schoendube FA. Influence of eNOS gene polymorphisms (894G/T;- 786C/T) on postoperative hemodynamics after cardiac surgery. The Thoracic and cardiovascular surgeon. 2006;54(4):233–8. Epub 2006/06/07. 10.1055/s-2005-873012 . 16755443

[19] Tzvetkov MV, Meineke I, Sehrt D, Vormfelde SV, Brockmoller J. Amelogenin-based sex identification as a strategy to control the identity of DNA samples in genetic association studies. Pharmacogenomics. 2010;11(3):449–57. Epub 2010/03/20. 10.2217/pgs.10.14 . 20235797

[20] Stephens M, Scheet P. Accounting for decay of linkage disequilibrium in haplotype inference and missing-data imputation. American journal of human genetics. 2005;76(3):449–62. Epub 2005/02/09. 10.1086/428594 15700229 PMC1196397

[21] Stevens PA, Brown MJ. Genetic variability of the ET-1 and the ETA receptor genes in essential hypertension. Journal of cardiovascular pharmacology. 1995;26 Suppl 3:S9–12. Epub 1995/01/01. . 8587478

[22] Nashef SA, Roques F, Hammill BG, Peterson ED, Michel P, Grover FL, et al Validation of European System for Cardiac Operative Risk Evaluation (EuroSCORE) in North American cardiac surgery. European journal of cardio-thoracic surgery: official journal of the European Association for Cardio-thoracic Surgery. 2002;22(1):101–5. Epub 2002/07/10. . 12103381 10.1016/s1010-7940(02)00208-7

[23] Vincent JL, Moreno R. Clinical review: scoring systems in the critically ill. Critical care (London, England). 2010;14(2):207 Epub 2010/04/16. 10.1186/cc8204 20392287 PMC2887099

[24] Frey UH, Muehlschlegel JD, Ochterbeck C, Fox AA, Shernan SK, Collard CD, et al GNAS gene variants affect beta-blocker-related survival after coronary artery bypass grafting. Anesthesiology. 2014;120(5):1109–17. Epub 2014/04/24. 10.1097/aln.0000000000000189 24755784 PMC4070180

[25] Osswald BR, Blackstone EH, Tochtermann U, Thomas G, Vahl CF, Hagl S. The meaning of early mortality after CABG. European journal of cardio-thoracic surgery: official journal of the European Association for Cardio-thoracic Surgery. 1999;15(4):401–7. Epub 1999/06/17. . 10371112 10.1016/s1010-7940(99)00029-9

[26] Podgoreanu MV, White WD, Morris RW, Mathew JP, Stafford-Smith M, Welsby IJ, et al Inflammatory gene polymorphisms and risk of postoperative myocardial infarction after cardiac surgery. Circulation. 2006;114(1 Suppl):I275–81. Epub 2006/07/06. 10.1161/circulationaha.105.001032 16820586 PMC1945056

