# Supplementary material for: Genetic Polymorphisms in Endothelin-1 as Predictors for Long-Term Survival and the Cardiac Index in Patients Undergoing On-Pump Cardiac Surgery
Source: PLoS One. 2015 Jun 29;10(6):e0131155. doi: 10.1371/journal.pone.0131155 (PMC4487899; doi:10.1371/journal.pone.0131155)
Supplement: S1 Tables — COPD: chronic obstructive pulmonary disease; ACE: angiotensin-converting enzyme; CABG: coronary artery bypass grafting. (DOCX) [file pone.0131155.s001.docx]

**Baseline characteristics, *EDN1* 1370TG (rs1800541)**

| **Variable** | **TT**  (n=311) | **GT**  (n=130) | **GG**  (n=14) | ***p* value** | ***p* value (F-test)** |
| --- | --- | --- | --- | --- | --- |
| Age (years) | 68±10 | 66±10 | 66±9 | 0.2247 | 0.3715 |
| Male/female (%) | 64.6/35.4 | 65.4/34.6 | 71.4/28.6 | 0.8688 |  |
| ***Risk factors*** |  |  |  |  |  |
| Body mass index (kg/m²) | 28±4 | 29±16 | 27±7 | 0.6394 | 0.3030 |
| Smoking (%) | 36.7 | 33.9 | 35.7 | 0.8543 |  |
| Hypertension (%) | 73.0 | 75.4 | 71.4 | 0.8578 |  |
| Hypercholesterolemia (%) | 47.3 | 54.6 | 21.4 | 0.0441 |  |
| Diabetes mellitus (%) | 32.8 | 29.2 | 35.7 | 0.7277 |  |
| Positive family history (%) | 18.0 | 11.5 | 28.6 | 0.1153 |  |
| Ejection fraction (%), (n) | 57±10 (114) | 55±11 (42) | 69±1 (2) | 0.0903 | 0.1640 |
| Renal disorder (%) | 12.9 | 16.9 | 7.1 | 0.4041 |  |
| Peripheral disease (%) | 6.1 | 10.8 | 0.0 | 0.1294 |  |
| Neurocerebral events (%) | 11.3 | 14.6 | 7.1 | 0.5201 |  |
| Pulmonary hypertension (%) | 6.1 | 12.3 | 7.1 | 0.0886 |  |
| COPD (%) | 8.7 | 5.4 | 14.3 | 0.3373 |  |
| Dialyisis (%) | 1.0 | 0.0 | 0.0 | 0.4970 |  |
| ***Preoperative medications*** |  |  |  |  |  |
| ß-blockers (%) | 65.3 | 65.4 | 71.4 | 0.8933 |  |
| ACE inhibitors (%) | 57.9 | 66.9 | 57.1 | 0.2016 |  |
| Oral nitrates (%) | 19.3 | 16.9 | 14.3 | 0.7752 |  |
| Antiarrhythmics (%) | 3.5 | 3.9 | 7.1 | 0.7825 |  |
| Diuretics (%) | 40.5 | 44.6 | 35.7 | 0.6581 |  |
| Antilipid agent (%) | 55.6 | 61.5 | 64.3 | 0.4544 |  |
| Antidiabetic (%) | 18.3 | 20.0 | 42.9 | 0.0763 |  |
| Antihypertensive agents (%) | 23.2 | 20.8 | 7.1 | 0.3400 |  |
| Bronchdilatotor (%) | 2.3 | 3.1 | 7.1 | 0.5001 |  |
| Anticoagulation (%) | 68.5 | 73.1 | 78.6 | 0.4930 |  |
| ***Urgency of surgery*** |  |  |  | 0.7794 |  |
| Elective (n = 378) (%) | 83.0 | 83.9 | 78.6 |  |  |
| Urgent (n = 40) (%) | 8.4 | 10.0 | 7.1 |  |  |
| Emergency (n = 37) (%) | 8.7 | 6.2 | 14.3 |  |  |
| ***Associated surgical procedures*** |  |  |  |  |  |
| CABG (n = 226) (%) | 49.2 | 53.1 | 28.6 | 0.2096 |  |
| Valve (n = 87) (%) | 17.7 | 20.8 | 35.7 | 0.2084 |  |
| Combined procedures (n = 90) (%) | 20.9 | 16.2 | 28.6 | 0.3669 |  |
| Other procedures (n = 52) (%) | 12.2 | 10.0 | 7.1 | 0.7019 |  |
| ***Euroscore additive*** | 5.6±4.0 | 5.3±3.4 | 5.6±3.9 | 0.9364 | 0.7621 |

Table legend: COPD: chronic obstructive pulmonary disease; ACE: angiotensin-converting enzyme; CABG: coronary artery bypass grafting. The column headings 0, 1 and 2 refer to the frequency of the haplotype; 0 means the haplotype does not exist, 1 means the haplotype appears once and 2 indicates that the haplotype exists twice.

**Baseline characteristics, *EDN1* K198N (rs5370)**

| **Variable** | **GG**  (n=267) | **GT**  (n=170) | **TT**  (n=18) | ***p* value** | ***p* value (F-test)** |
| --- | --- | --- | --- | --- | --- |
| Age (years) | 68±10 | 66±11 | 67±8 | 0.0617 | 0.0343 |
| Male/female (%) | 64.8/35.2 | 65.3/34.7 | 66.7/33.3 | 0.9837 |  |
| ***Risk factors*** |  |  |  |  |  |
| Body mass index (kg/m²) | 28±4 | 29±14 | 27±5 | 0.5941 | 0.3464 |
| Smoking (%) | 35.6 | 36.5 | 33.3 | 0.9577 |  |
| Hypertension (%) | 72.7 | 73.5 | 88.9 | 0.3184 |  |
| Hypercholesterolemia (%) | 46.4 | 52.4 | 44.4 | 0.4537 |  |
| Diabetes mellitus (%) | 34.1 | 28.8 | 27.8 | 0.4801 |  |
| Positive family history (%) | 18.0 | 12.9 | 27.8 | 0.1611 |  |
| Ejection fraction (%), (n) | 57±10 (100) | 56±11 (56) | 59±13 (2) | 0.9672 | 0.9364 |
| Renal disorder (%) | 12.0 | 17.7 | 5.6 | 0.1443 |  |
| Peripheral disease (%) | 6.0 | 9.4 | 5.6 | 0.3895 |  |
| Neurocerebral events (%) | 10.9 | 14.1 | 11.1 | 0.5906 |  |
| Pulmonary hypertension (%) | 6.4 | 10.6 | 5.6 | 0.2614 |  |
| COPD (%) | 7.5 | 8.2 | 11.1 | 0.8427 |  |
| Dialyisis (%) | 1.1 | 0.0 | 0.0 | 0.3453 |  |
| ***Preoperative medications*** |  |  |  |  |  |
| ß-blockers (%) | 65.2 | 65.3 | 72.2 | 0.8285 |  |
| ACE inhibitors (%) | 57.7 | 64.7 | 61.1 | 0.3414 |  |
| Oral nitrates (%) | 18.4 | 19.4 | 11.1 | 0.6872 |  |
| Antiarrhythmics (%) | 4.1 | 2.9 | 5.6 | 0.7506 |  |
| Diuretics (%) | 38.2 | 47.7 | 33.3 | 0.1144 |  |
| Antilipid agent (%) | 55.1 | 60.0 | 72.2 | 0.2613 |  |
| Antidiabetic (%) | 18.7 | 20.0 | 27.8 | 0.6340 |  |
| Antihypertensive agents (%) | 22.9 | 20.6 | 22.2 | 0.8566 |  |
| Bronchdilatotor (%) | 2.6 | 2.4 | 5.6 | 0.7222 |  |
| Anticoagulation (%) | 68.2 | 71.2 | 88.9 | 0.1650 |  |
| ***Urgency of surgery*** |  |  |  | 0.6628 |  |
| Elective (n = 378) (%) | 83.9 | 81.8 | 83.3 |  |  |
| Urgent (n = 40) (%) | 7.5 | 11.2 | 5.6 |  |  |
| Emergency (n = 37) (%) | 8.6 | 7.1 | 11.1 |  |  |
| ***Associated surgical procedures*** |  |  |  |  |  |
| CABG (n = 226) (%) | 47.6 | 54.1 | 38.9 | 0.2651 |  |
| Valve (n = 87) (%) | 18.0 | 20.0 | 27.8 | 0.5535 |  |
| Combined procedures (n = 90) (%) | 20.2 | 18.8 | 22.2 | 0.9053 |  |
| Other procedures (n = 52) (%) | 14.2 | 7.1 | 11.1 | 0.0712 |  |
| ***Euroscore additive*** | 5.8±4.1 | 5.1±3.4 | 4.9±3.7 | 0.3251 | 0.2106 |

Table legend: COPD: chronic obstructive pulmonary disease; ACE: angiotensin-converting enzyme; CABG: coronary artery bypass grafting. The column headings 0, 1 and 2 refer to the frequency of the haplotype; 0 means the haplotype does not exist, 1 means the haplotype appears once and 2 indicates that the haplotype exists twice.

**Baseline characteristics, *ET1*-H1 (T1370G=T; K198N=G)**

| **Variable** | **0**  (n=21) | **1**  (n=172) | **2**  (n=262) | ***p* value** | ***p* value (F-test)** |
| --- | --- | --- | --- | --- | --- |
| Age (years) | 67±8 | 66±11 | 68±10 | 0.1141 | 0.0680 |
| Male/female (%) | 71.4/28.6 | 63.4/36.6 | 65.7/34.4 | 0.7297 |  |
| ***Risk factors*** |  |  |  |  |  |
| Body mass index (kg/m²) | 27±6 | 29±14 | 28±4 | 0.8004 | 0.3879 |
| Smoking (%) | 33.3 | 36.6 | 35.5 | 0.9430 |  |
| Hypertension (%) | 81.0 | 73.8 | 72.9 | 0.7205 |  |
| Hypercholesterolemia (%) | 38.1 | 52.9 | 46.6 | 0.2672 |  |
| Diabetes mellitus (%) | 28.6 | 27.9 | 34.7 | 0.3106 |  |
| Positive family history (%) | 23.8 | 14.0 | 17.6 | 0.3989 |  |
| Ejection fraction (%), (n) | 63±11 (3) | 56±11 (57) | 57±11(98) | 0.5493 | 0.5702 |
| Renal disorder (%) | 4.8 | 17.4 | 12.2 | 0.1421 |  |
| Peripheral disease (%) | 4.8 | 9.3 | 6.1 | 0.4108 |  |
| Neurocerebral events (%) | 9.5 | 14.0 | 11.1 | 0.6220 |  |
| Pulmonary hypertension (%) | 4.8 | 11.1 | 6.1 | 0.1512 |  |
| COPD (%) | 9.5 | 8.1 | 7.6 | 0.9441 |  |
| Dialyisis (%) | 0.0 | 0.0 | 1.2 | 0.3288 |  |
| ***Preoperative medications*** |  |  |  |  |  |
| ß-blockers (%) | 71.4 | 66.3 | 64.5 | 0.7835 |  |
| ACE inhibitors (%) | 61.9 | 65.1 | 57.3 | 0.2585 |  |
| Oral nitrates (%) | 9.5 | 19.8 | 18.3 | 0.5187 |  |
| Antiarrhythmics (%) | 4.8 | 2.9 | 4.2 | 0.7611 |  |
| Diuretics (%) | 33.3 | 47.7 | 38.2 | 0.1067 |  |
| Antilipid agent (%) | 66.7 | 59.9 | 55.3 | 0.4448 |  |
| Antidiabetic (%) | 28.6 | 19.2 | 19.1 | 0.5664 |  |
| Antihypertensive agents (%) | 19.1 | 20.4 | 23.3 | 0.7292 |  |
| Bronchdilatotor (%) | 4.8 | 2.3 | 2.7 | 0.8043 |  |
| Anticoagulation (%) | 81.0 | 71.5 | 68.3 | 0.4190 |  |
| ***Urgency of surgery*** |  |  |  | 0.6807 |  |
| Elective (n = 378) (%) | 85.7 | 82.0 | 83.6 |  |  |
| Urgent (n = 40) (%) | 4.8 | 11.1 | 7.6 |  |  |
| Emergency (n = 37) (%) | 9.5 | 7.0 | 8.8 |  |  |
| ***Associated surgical procedures*** |  |  |  |  |  |
| CABG (n = 226) (%) | 33.3 | 54.7 | 47.7 | 0.1135 |  |
| Valve (n = 87) (%) | 33.3 | 19.8 | 17.6 | 0.2015 |  |
| Combined procedures (n = 90) (%) | 23.8 | 18.0 | 20.6 | 0.7177 |  |
| Other procedures (n = 52) (%) | 9.5 | 7.6 | 14.1 | 0.1054 |  |
| ***Euroscore additive*** | 4.9±3.5 | 5.2±3.4 | 5.7±4.1 | 0.3845 | 0.2462 |

Table legend: COPD: chronic obstructive pulmonary disease; ACE: angiotensin-converting enzyme; CABG: coronary artery bypass grafting. The column headings 0, 1 and 2 refer to the frequency of the haplotype; 0 means the haplotype does not exist, 1 means the haplotype appears once and 2 indicates that the haplotype exists twice.

**Baseline characteristics, *ET1*-H2 (T1370G=G; K198N=T)**

| **Variable** | **0**  (n=316) | **1**  (n=128) | **2**  (n=11) | ***p* value** | ***p* value (F-test)** |
| --- | --- | --- | --- | --- | --- |
| Age (years) | 68±10 | 66±10 | 66±9 | 0.1280 | 0.2403 |
| Male/female (%) | 63.5/36.5 | 67.4/32.6 | 63.6/36.4 | 0.7258 |  |
| ***Risk factors*** |  |  |  |  |  |
| Body mass index (kg/m²) | 28±4 | 29±16 | 26±6 | 0.3634 | 0.2598 |
| Smoking (%) | 36.7 | 33.6 | 36.4 | 0.8245 |  |
| Hypertension (%) | 72.8 | 75.0 | 81.8 | 0.7335 |  |
| Hypercholesterolemia (%) | 47.2 | 53.9 | 27.3 | 0.1563 |  |
| Diabetes mellitus (%) | 32.3 | 30.5 | 36.4 | 0.8858 |  |
| Positive family history (%) | 18.4 | 10.2 | 36.4 | 0.0214 |  |
| Ejection fraction (%), (n) | 57±10 (116) | 56±11 (41) | 68±0 (1) | 0.3438 | 0.4394 |
| Renal disorder (%) | 12.7 | 17.2 | 9.1 | 0.4105 |  |
| Peripheral disease (%) | 6.0 | 10.9 | 0.0 | 0.1245 |  |
| Neurocerebral events (%) | 11.1 | 14.8 | 9.1 | 0.5188 |  |
| Pulmonary hypertension (%) | 6.3 | 11.7 | 9.1 | 0.1609 |  |
| COPD (%) | 8.5 | 5.5 | 18.2 | 0.2448 |  |
| Dialyisis (%) | 1.0 | 0.0 | 0.0 | 0.5146 |  |
| ***Preoperative medications*** |  |  |  |  |  |
| ß-blockers (%) | 65.8 | 64.1 | 72.7 | 0.8245 |  |
| ACE inhibitors (%) | 58.2 | 66.4 | 54.6 | 0.2576 |  |
| Oral nitrates (%) | 19.3 | 16.4 | 18.2 | 0.7754 |  |
| Antiarrhythmics (%) | 3.5 | 3.9 | 9.1 | 0.6236 |  |
| Diuretics (%) | 40.5 | 44.5 | 36.4 | 0.6934 |  |
| Antilipid agent (%) | 55.4 | 61.7 | 72.7 | 0.2784 |  |
| Antidiabetic (%) | 18.0 | 21.1 | 45.5 | 0.0691 |  |
| Antihypertensive agents (%) | 22.8 | 21.1 | 9.1 | 0.5369 |  |
| Bronchdilatotor (%) | 2.2 | 3.1 | 9.1 | 0.3461 |  |
| Anticoagulation (%) | 68.4 | 72.7 | 90.9 | 0.2089 |  |
| ***Urgency of surgery*** |  |  |  | 0.6405 |  |
| Elective (n = 378) (%) | 83.2 | 83.6 | 72.7 |  |  |
| Urgent (n = 40) (%) | 8.2 | 10.2 | 9.1 |  |  |
| Emergency (n = 37) (%) | 8.5 | 6.3 | 18.2 |  |  |
| ***Associated surgical procedures*** |  |  |  |  |  |
| CABG (n = 226) (%) | 49.1 | 52.3 | 36.4 | 0.5505 |  |
| Valve (n = 87) (%) | 18.0 | 21.1 | 27.3 | 0.5961 |  |
| Combined procedures (n = 90) (%) | 20.6 | 17.2 | 27.3 | 0.5899 |  |
| Other procedures (n = 52) (%) | 12.3 | 9.4 | 9.1 | 0.6527 |  |
| ***Euroscore additive*** | 5.6±4.0 | 5.2±3.4 | 5.9±4.2 | 0.7888 | 0.6069 |

Table legend: COPD: chronic obstructive pulmonary disease; ACE: angiotensin-converting enzyme; CABG: coronary artery bypass grafting. The column headings 0, 1 and 2 refer to the frequency of the haplotype; 0 means the haplotype does not exist, 1 means the haplotype appears once and 2 indicates that the haplotype exists twice.

**Baseline characteristics, *ET1*-H3 (T1370G=T; K198N=T)**

| **Variable** | **0**  (n=399) | **1**  (n=56) | **2**  (n=0) | ***p* value** | ***p* value (F-test)** |
| --- | --- | --- | --- | --- | --- |
| Age (years) | 68±10 | 66±12 |  | 0.5525 | 0.1998 |
| Male/female (%) | 65.7/34.3 | 60.7/39.3 |  | 0.4669 |  |
| ***Risk factors*** |  |  |  |  |  |
| Body mass index (kg/m²) | 28±10 | 28±4 |  | 0.7912 | 0.8927 |
| Smoking (%) | 35.1 | 41.1 |  | 0.3818 |  |
| Hypertension (%) | 73.2 | 76.8 |  | 0.5666 |  |
| Hypercholesterolemia (%) | 47.9 | 53.6 |  | 0.4240 |  |
| Diabetes mellitus (%) | 33.3 | 21.4 |  | 0.0733 |  |
| Positive family history (%) | 16.0 | 19.6 |  | 0.4962 |  |
| Ejection fraction (%), (n) | 56±11 (141) | 58±10 (17) |  | 0.5420 | 0.6461 |
| Renal disorder (%) | 13.8 | 14.3 |  | 0.9189 |  |
| Peripheral disease (%) | 7.3 | 7.1 |  | 0.9729 |  |
| Neurocerebral events (%) | 12.0 | 12.5 |  | 0.9195 |  |
| Pulmonary hypertension (%) | 8.3 | 5.4 |  | 0.4494 |  |
| COPD (%) | 7.3 | 12.5 |  | 0.1743 |  |
| Dialyisis (%) | 0.8 | 0.0 |  | 0.5150 |  |
| ***Preoperative medications*** |  |  |  |  |  |
| ß-blockers (%) | 64.9 | 69.6 |  | 0.4855 |  |
| ACE inhibitors (%) | 60.2 | 62.5 |  | 0.7363 |  |
| Oral nitrates (%) | 18.1 | 21.4 |  | 0.5411 |  |
| Antiarrhythmics (%) | 4.3 | 0.0 |  | 0.1154 |  |
| Diuretics (%) | 40.4 | 50.0 |  | 0.1700 |  |
| Antilipid agent (%) | 57.4 | 58.9 |  | 0.8276 |  |
| Antidiabetic (%) | 20.6 | 12.5 |  | 0.1549 |  |
| Antihypertensive agents (%) | 21.6 | 25.0 |  | 0.5597 |  |
| Bronchdilatotor (%) | 3.0 | 0.0 |  | 0.1884 |  |
| Anticoagulation (%) | 69.9 | 71.4 |  | 0.8179 |  |
| ***Urgency of surgery*** |  |  |  | 0.8385 |  |
| Elective (n = 378) (%) | 83.2 | 82.1 |  |  |  |
| Urgent (n = 40) (%) | 8.5 | 10.7 |  |  |  |
| Emergency (n = 37) (%) | 8.3 | 7.1 |  |  |  |
| ***Associated surgical procedures*** |  |  |  |  |  |
| CABG (n = 226) (%) | 48.9 | 55.4 |  | 0.3633 |  |
| Valve (n = 87) (%) | 19.1 | 19.6 |  | 0.9155 |  |
| Combined procedures (n = 90) (%) | 19.6 | 21.4 |  | 0.7408 |  |
| Other procedures (n = 52) (%) | 12.5 | 3.6 |  | 0.0484 |  |
| ***Euroscore additive*** | 5.6±3.9 | 4.5±3.3 |  | 0.0387 | 0.0425 |

Table legend: COPD: chronic obstructive pulmonary disease; ACE: angiotensin-converting enzyme; CABG: coronary artery bypass grafting. The column headings 0, 1 and 2 refer to the frequency of the haplotype; 0 means the haplotype does not exist, 1 means the haplotype appears once and 2 indicates that the haplotype exists twice.

**Baseline characteristics, *ET1*-H4 (T1370G=G; K198N=G)**

| **Variable** | **0**  (n=447) | **1**  (n=8) | **2**  (n=0) | ***p* value** | ***p* value (F-test)** |
| --- | --- | --- | --- | --- | --- |
| Age (years) | 67±10 | 70±8 |  | 0.3448 | 0.3961 |
| Male/female (%) | 65.3/34.7 | 50.0/50.0 |  | 0.3675 |  |
| ***Risk factors*** |  |  |  |  |  |
| Body mass index (kg/m²) | 28±9 | 27±6 |  | 0.8250 | 0.7549 |
| Smoking (%) | 35.8 | 37.5 |  | 0.9205 |  |
| Hypertension (%) | 74.1 | 50.0 |  | 0.1260 |  |
| Hypercholesterolemia (%) | 49.0 | 25.0 |  | 0.1783 |  |
| Diabetes mellitus (%) | 67.8 | 87.5 |  | 0.2355 |  |
| Positive family history (%) | 16.3 | 25.0 |  | 0.5124 |  |
| Ejection fraction (%), (n) | 57±11 (155) | 60±10 (3) |  | 0.6171 | 0.5687 |
| Renal disorder (%) | 14.1 | 0.0 |  | 0.2526 |  |
| Peripheral disease (%) | 7.4 | 0.0 |  | 0.4248 |  |
| Neurocerebral events (%) | 12.3 | 0.0 |  | 0.2899 |  |
| Pulmonary hypertension (%) | 7.8 | 12.5 |  | 0.6276 |  |
| COPD (%) | 8.1 | 0.0 |  | 0.4029 |  |
| Dialyisis (%) | 1.0 | 0.0 |  | 0.8161 |  |
| ***Preoperative medications*** |  |  |  |  |  |
| ß-blockers (%) | 65.1 | 87.5 |  | 0.1865 |  |
| ACE inhibitors (%) | 60.2 | 75.0 |  | 0.3954 |  |
| Oral nitrates (%) | 18.6 | 12.5 |  | 0.6610 |  |
| Antiarrhythmics (%) | 3.8 | 0.0 |  | 0.5739 |  |
| Diuretics (%) | 41.6 | 37.5 |  | 0.8150 |  |
| Antilipid agent (%) | 57.9 | 37.5 |  | 0.2462 |  |
| Antidiabetic (%) | 19.7 | 12.5 |  | 0.6115 |  |
| Antihypertensive agents (%) | 22.4 | 0.0 |  | 0.1298 |  |
| Bronchdilatotor (%) | 2.7 | 0.0 |  | 0.6385 |  |
| Anticoagulation (%) | 70.5 | 50.0 |  | 0.2099 |  |
| ***Urgency of surgery*** |  |  |  | 0.4363 |  |
| Elective (n = 378) (%) | 82.8 | 100.0 |  |  |  |
| Urgent (n = 40) (%) | 9.0 | 0.0 |  |  |  |
| Emergency (n = 37) (%) | 8.3 | 0.0 |  |  |  |
| ***Associated surgical procedures*** |  |  |  |  |  |
| CABG (n = 226) (%) | 50.1 | 25.0 |  | 0.1591 |  |
| Valve (n = 87) (%) | 18.6 | 50.0 |  | 0.0250 |  |
| Combined procedures (n = 90) (%) | 19.9 | 12.5 |  | 0.6019 |  |
| Other procedures (n = 52) (%) | 11.4 | 12.5 |  | 0.9234 |  |
| ***Euroscore additive*** | 5.5±3.8 | 5.6±3.0 |  | 0.7345 | 0.9171 |

Table legend: COPD: chronic obstructive pulmonary disease; ACE: angiotensin-converting enzyme; CABG: coronary artery bypass grafting. The column headings 0, 1 and 2 refer to the frequency of the haplotype; 0 means the haplotype does not exist, 1 means the haplotype appears once and 2 indicates that the haplotype exists twice.
