# Supplementary material for: Genetic Polymorphisms in Endothelin-1 as Predictors for Long-Term Survival and the Cardiac Index in Patients Undergoing On-Pump Cardiac Surgery
Source: PLoS One. 2015 Jun 29;10(6):e0131155. doi: 10.1371/journal.pone.0131155 (PMC4487899; doi:10.1371/journal.pone.0131155)
Supplement: S2 Tables — PaO2/FiO2: oxygenation index; PEEP: positive end-expiratory pressure; PCO2: partial pressure of carbon dioxide; APACHE II Score: Acute Physiology and Chronic Health Evaluation Score; SAPS II Score: Simplified Acute Physiology Score; HR: Heart rate; MAP: mean arterial pressure; CVP: central venous pressure; PCWP: pulmonary capillary wedge pressure; PAP: mean pulmonary artery pressure; CI: cardiac index; SVRI: systemic vascular resistance; PVRI: pulmonary vascular resistance; NTG: nitroglycerin; IABP: intraaortic balloon pump; ECMO: extracorporeal membrane oxygenation; ICU: intensive care unit. (DOCX) [file pone.0131155.s002.docx]

**Peri- and postoperative course associated with *EDN1* 1370TG (rs1800541)**

| **Variable** | **TT**  (n=311) | **GT**  (n=130) | **GG**  (n=14) | ***p* value** | ***p* value (F-test)** |
| --- | --- | --- | --- | --- | --- |
| ***Pulmonary function*** |  |  |  |  |  |
| P_a_O_2_/F_i_O_2_ | 261±83 | 248±64 | 262±66 | 0.5536 | 0.2758 |
| PEEP (mbar) | 7±2 | 7±1 | 6±0 | 0.0953 | 0.0572 |
| PCO_2_ (mmHg) | 40±4 | 40±4 | 40±4 | 0.7818 | 0.8206 |
| Arterial pH | 7.40±0.04 | 7.40±0.04 | 7.39±0.03 | 0.2529 | 0.3057 |
| Compliance (ml/mbar) | 47±24 | 47±17 | 53±17 | 0.2680 | 0.5897 |
| Infiltrates (quadrants) | 1.29±0.63 | 1.17±0.67 | 0.98±0.59 | 0.0468 | 0.0549 |
| Lung injury score | 1±1 | 1±1 | 1±1 | 0.1770 | 0.1602 |
| ***Scores*** |  |  |  |  |  |
| APACHE II Score | 14.9±6.4 | 14.5±6.6 | 17.2±8.4 | 0.4293 | 0.3311 |
| SAPS II Score | 25.0±8.0 | 23.9±5.9 | 22.1±6.0 | 0.1033 | 0.1416 |
| ***Hemodynamic*** |  |  |  |  |  |
| Heart rate (bpm) | 82±10 | 85±10 | 81±7 | 0.0040 | 0.0033 |
| MAP (mmHg) | 81±7 | 82±7 | 84±7 | 0.2306 | 0.1563 |
| CVP (mmHg) | 11±2 | 12±3 | 11±2 | 0.1113 | 0.1072 |
| PCWP (mmHg), (n) | 15±4 (78) | 15±3 (40) | 10±0 (1) | 0.2404 | 0.2365 |
| PAP sys (mmHg), (n) | 37±8 (87) | 36±9 (47) | 29±0 (1) | 0.2770 | 0.4842 |
| PAP dia (mmHg), (n) | 19±5 (87) | 18±5 (47) | 16±0 (1) | 0.6827 | 0.7183 |
| PAP mean (mmHg), (n) | 26±8 (101) | 26±9 (51) | 28±22 (3) | 0.6224 | 0.9047 |
| CI (l/min/m²), (n) | 3±1 (93) | 3±1 (45) | 2±0 (1) | 0.0062 | 0.0553 |
| SVRI (dyn∙s^-1^∙m²∙cm^-5^), (n) | 1027±504 (86) | 890±215 (39) | 1015±0 (1) | 0.2544 | 0.2700 |
| PVRI (dyn∙s^-1^∙m²∙cm^-5^), (n) | 223±127 (74) | 211±162 (38) | 120±0 (1) | 0.1878 | 0.7142 |
| ***Renal system*** |  |  |  |  |  |
| Urine output (ml/d) | 1816±782 | 1810±734 | 2103±761 | 0.4909 | 0.3828 |
| Urine output (ml/kg/hr) | 1±1 | 1±1 | 1±0 | 0.3145 | 0.4365 |
| Serum creatinine (mg/dl) | 1.3±0.6 | 1.4±1.1 | 1.3±0.5 | 0.9262 | 0.5369 |
| Serum urea (mg/dl) | 27.2±13.1 | 28.5±14.3 | 22.6±9.3 | 0.3966 | 0.2499 |
| Creatinine clearance | 69.0±31.6 | 70.9±31.0 | 71.7±26.9 | 0.5835 | 0.8130 |
| ***Inotropes*** |  |  |  |  |  |
| Epinephrine (mg/d), (n) | 1.1±5.0 (244) | 2.5±16.5 (101) | 0.1±0.2 (10) | 0.3153 | 0.3634 |
| Norepinephrine (mg/d), (n) | 1.0±3.4 (164) | 1.3±5.0 (80) | 0.1±0.1 (6) | 0.0775 | 0.5074 |
| Enoximone (mg/d), (n) | 8.3±45.4 (43) | 9.6±55.8 (20) | 0.0±0.0 (0) | 0.2932 | 0.7733 |
| Dobutamine (mg/d), (n) | 14.3±47.5 (64) | 10.8±48.1 (23) | 2.0±6.7 (2) | 0.6157 | 0.5311 |
| ***Other agents*** |  |  |  |  |  |
| NTG (mg/d), (n) | 5.2±6.6 (233) | 5.0±6.6 (99) | 4.6±6.3 (7) | 0.6554 | 0.8986 |
| Amiodarone (mg/d), (n) | 72.7±241.2 (89) | 67.6±220.2 (37) | 6.1±21.4 (2) | 0.4040 | 0.5727 |
| Cortisone (mg/d), (n) | 34.0±178.5 (34) | 22.6±120.7 (17) | 0.0±0.0 (0) | 0.3454 | 0.6223 |
| Vasopressin (iU/d), (n) | 0.1±0.7 (8) | 0.1±0.8 (4) | 0.0±0.0 (0) | 0.7869 | 0.8750 |
| Furosemide (mg/d), (n) | 37.8±72.5 (277) | 33.8±57.0 (122) | 22.6±19.9 (14) | 0.4464 | 0.6335 |
| ***Operative characteristics*** |  |  |  |  |  |
| Cross-clamp time (min) | 94±38 | 94±38 | 109±33 | 0.1906 | 0.3653 |
| Cardiopulmonary bypass time (min) | 145±73 | 143±59 | 165±53 | 0.1904 | 0.5196 |
| Transfused red blood cells (ml/d) | 658±717 | 842±904 | 576±654 | 0.1541 | 0.0608 |
| Fresh frozen plasma (ml/d) | 68±264 | 98±261 | 37±139 | 0.1581 | 0.4798 |
| Prothrombin complex concentrates (iU/d) | 8±84 | 20±137 | 0±0 | 0.3713 | 0.5112 |
| IABP (%) | 6.1 | 6.2 | 0 | 0.6339 |  |
| ECMO (%) | 0 | 0 | 7.1 | 0.0005 |  |
| Length of ICU stay (d) | 7±14 | 7±15 | 6±12 | 0.6064 | 0.9151 |
| Hospitality stay (d) | 25±21 | 25±19 | 20±10 | 0.8514 | 0.6978 |
| ***In-hospital mortality (n=36)*** |  |  |  | 0.5227 |  |
| Overall (7.9%) (%), n= 455 | 8.4 | 7.7 | 0.0 |  |  |
| Elective cases (%), n=378 | 5.5 | 6.2 | 0.0 |  |  |
| Urgent cases (%), n=40 | 1.9 | 0.8 | 0.0 |  |  |
| Emergency cases (%), n=37 | 1.0 | 0.8 | 0.0 |  |  |
| Age at surgery (years) | 68±10 | 66±10 | 66±9 | 0.2247 | 0.3715 |
| Survival after surgery (days) | 516±544 | 527±553 | 576±0 | 0.8782 | 0.9902 |
| Age at death (years) | 74±7 | 72±7 | 67±0 | 0.3199 | 0.4657 |

Table legend: P_a_O_2_/F_i_O_2_: oxygenation index; PEEP: positive end-expiratory pressure; PCO_2_: partial pressure of carbon dioxide; APACHE II Score: Acute Physiology and Chronic Health Evaluation Score; SAPS II Score: Simplified Acute Physiology Score; HR: Heart rate; MAP: mean arterial pressure; CVP: central venous pressure; PCWP: pulmonary capillary wedge pressure; PAP: mean pulmonary artery pressure; CI: cardiac index; SVRI: systemic vascular resistance; PVRI: pulmonary vascular resistance; NTG: nitroglycerin; IABP: intraaortic balloon pump; ECMO: extracorporeal membrane oxygenation; ICU: intensive care unit. The column headings 0, 1 and 2 refer to the frequency of the haplotype; 0 means the haplotype does not exist, 1 means the haplotype appears once and 2 indicates that the haplotype exists twice.**Peri- and postoperative course associated with *EDN1* K198N (rs5370)**

| **Variable** | **GG**  (n=267) | **GT**  (n=170) | **TT**  (n=18) | ***p* value** | ***p* value (F-test)** |
| --- | --- | --- | --- | --- | --- |
| ***Pulmonary function*** |  |  |  |  |  |
| P_a_O_2_/F_i_O_2_ | 258±82 | 255±72 | 264±62 | 0.7365 | 0.8218 |
| PEEP (mbar) | 7±1 | 7±2 | 7±1 | 0.6813 | 0.6581 |
| PCO_2_ (mmHg) | 40±3 | 40±4 | 39±4 | 0.6417 | 0.6753 |
| Arterial pH | 7.40±0.03 | 7.40±0.04 | 7.40±0.03 | 0.7261 | 0.7367 |
| Compliance (ml/mbar) | 48±26 | 47±17 | 47±15 | 0.9564 | 0.9462 |
| Infiltrates (quadrants) | 1.30±0.63 | 1.17±0.67 | 1.19±0.60 | 0.0965 | 0.1223 |
| Lung injury score | 1±1 | 1±1 | 1±1 | 0.3485 | 0.3914 |
| ***Scores*** |  |  |  |  |  |
| APACHE II Score | 15.0±6.3 | 14.5±6.9 | 14.8±6.4 | 0.4173 | 0.7104 |
| SAPS II Score | 25.2±7.8 | 23.7±6.9 | 23.8±4.9 | 0.0349 | 0.0933 |
| ***Hemodynamic*** |  |  |  |  |  |
| Heart rate (bpm) | 82±9 | 84±11 | 82±8 | 0.0833 | 0.0818 |
| MAP (mmHg) | 81±7 | 82±7 | 83±7 | 0.6565 | 0.4773 |
| CVP (mmHg) | 11±3 | 11±2 | 11±2 | 0.3824 | 0.4472 |
| PCWP (mmHg), (n) | 15±4 (71) | 15±3 (46) | 12±3 (2) | 0.3047 | 0.3091 |
| PAP sys (mmHg), (n) | 38±8 (79) | 36±9 (54) | 29±0 (2) | 0.0925 | 0.1982 |
| PAP dia (mmHg), (n) | 19±5 (79) | 18±5 (54) | 15±2 (2) | 0.2930 | 0.3108 |
| PAP mean (mmHg), (n) | 26±7 (91) | 26±10 (61) | 31±19 (3) | 0.4992 | 0.6329 |
| CI (l/min/m²), (n) | 3±1 (86) | 3±1 (51) | 3±1 (2) | 0.0081 | 0.0785 |
| SVRI (dyn∙s^-1^∙m²∙cm^-5^), (n) | 1037±521 (79) | 894±216 (46) | 1015±0 (1) | 0.1826 | 0.2079 |
| PVRI (dyn∙s^-1^∙m²∙cm^-5^), (n) | 220±129 (69) | 217±157 (43) | 120±0 (1) | 0.3499 | 0.7774 |
| ***Renal system*** |  |  |  |  |  |
| Urine output (ml/d) | 1868±799 | 1718±710 | 2156±697 | 0.0310 | 0.0237 |
| Urine output (ml/kg/hr) | 1±1 | 1±1 | 1±0 | 0.0160 | 0.0942 |
| Serum creatinine (mg/dl) | 1.4±0.6 | 1.4±1.0 | 1.2±0.5 | 0.3701 | 0.4583 |
| Serum urea (mg/dl) | 27.4±13.0 | 28.1±14.2 | 21.7±8.8 | 0.2144 | 0.1581 |
| Creatinine clearance | 68.0±31.3 | 71.8±31.7 | 72.9±24.2 | 0.2309 | 0.4092 |
| ***Inotropes*** |  |  |  |  |  |
| Epinephrine (mg/d), (n) | 1.0±4.7 (209) | 2.4±14.7 (133) | 0.1±0.2(13) | 0.2264 | 0.2755 |
| Norepinephrine (mg/d), (n) | 0.9±3.2 (146) | 1.4±4.9 (95) | 0.1±0.1 (9) | 0.3930 | 0.2313 |
| Enoximone (mg/d), (n) | 6.6±29.1 (39) | 12.1±69.3 (23) | 0.3±1.1 (1) | 0.5227 | 0.3791 |
| Dobutamine (mg/d), (n) | 14.1±47.3 (55) | 12.4±48.8 (33) | 0.1±0.6 (1) | 0.2592 | 0.4712 |
| ***Other agents*** |  |  |  |  |  |
| NTG (mg/d), (n) | 5.2±6.5 (203) | 5.2±6.8 (124) | 4.3±5.5 (12) | 0.7090 | 0.8464 |
| Amiodarone (mg/d), (n) | 74.3±248.8 (82) | 66.6±213.9 (44) | 18.5±77.3 (2) | 0.1568 | 0.6042 |
| Cortisone (mg/d), (n) | 28.7±162.7 (30) | 34.4±167.1 (21) | 0.0±0.0 (0) | 0.2868 | 0.6833 |
| Vasopressin (iU/d), (n) | 0.1±0.8 (7) | 0.1±0.7 (5) | 0.0±0.0 (0) | 0.7619 | 0.8615 |
| Furosemide (mg/d), (n) | 37.2±69.3 (239) | 36.3±67.4 (156) | 20.4±18.3 (18) | 0.6103 | 0.5914 |
| ***Operative characteristics*** |  |  |  |  |  |
| Cross-clamp time (min) | 94±39 | 94±37 | 99±35 | 0.7905 | 0.8819 |
| Cardiopulmonary bypass time (min) | 145±75 | 144±60 | 146±49 | 0.7228 | 0.9766 |
| Transfused red blood cells (ml/d) | 635±684 | 843±903 | 534±621 | 0.0602 | 0.0145 |
| Fresh frozen plasma (ml/d) | 59±255 | 104±275 | 58±168 | 0.0490 | 0.2071 |
| Prothrombin complex concentrates (iU/d) | 9±90 | 15±120 | 0±0 | 0.6710 | 0.7543 |
| IABP (%) | 6.4 | 5.9 | 0.0 | 0.5417 |  |
| ECMO (%) | 0.0 | 1.0 | 5.6 | 0.0024 |  |
| Length of ICU stay (d) | 7±14 | 7±15 | 7±14 | 0.9433 | 0.9956 |
| Hospitality stay (d) | 25±21 | 25±19 | 22±11 | 0.9952 | 0.8319 |
| ***In-hospital mortality (n=36)*** |  |  |  | 0.3436 |  |
| Overall (7.9%) (%), n=455 | 7.5 | 9.4 | 0.0 |  |  |
| Elective cases (%), n=378 | 4.9 | 7.1 | 0.0 |  |  |
| Urgent cases (%), n=40 | 1.5 | 1.8 | 0.0 |  |  |
| Emergency cases (%), n=37 | 1.1 | 0.6 | 0.0 |  |  |
| Age at surgery (years) | 68±10 | 66±11 | 67±8 | 0.0617 | 0.0343 |
| Survival after surgery (days) | 557±552 | 459±544 | 500±1130 | 0.5045 | 0.6542 |
| Age at death (years) | 74±7 | 72±8 | 71±5 | 0.3519 | 0.2960 |

Table legend: P_a_O_2_/F_i_O_2_: oxygenation index; PEEP: positive end-expiratory pressure; PCO_2_: partial pressure of carbon dioxide; APACHE II Score: Acute Physiology and Chronic Health Evaluation Score; SAPS II Score: Simplified Acute Physiology Score; HR: Heart rate; MAP: mean arterial pressure; CVP: central venous pressure; PCWP: pulmonary capillary wedge pressure; PAP: mean pulmonary artery pressure; CI: cardiac index; SVRI: systemic vascular resistance; PVRI: pulmonary vascular resistance; NTG: nitroglycerin; IABP: intraaortic balloon pump; ECMO: extracorporeal membrane oxygenation; ICU: intensive care unit. The column headings 0, 1 and 2 refer to the frequency of the haplotype; 0 means the haplotype does not exist, 1 means the haplotype appears once and 2 indicates that the haplotype exists twice.

**Peri- and postoperative course associated with *EDN1* H1 (T1370G=T; K198N=G)**

| **Variable** | **0**  (n=21) | **1**  (n=172) | **2**  (n=262) | ***p* value** | ***p* value (F-test)** |
| --- | --- | --- | --- | --- | --- |
| ***Pulmonary function*** |  |  |  |  |  |
| P_a_O_2_/F_i_O_2_ | 259±61 | 254±73 | 259±82 | 0.8869 | 0.7942 |
| PEEP (mbar) | 6±1 | 7±2 | 7±1 | 0.4708 | 0.4910 |
| PCO_2_ (mmHg) | 39±3 | 40±4 | 40±3 | 0.3429 | 0.4121 |
| Arterial pH | 7.40±0.03 | 7.40±0.04 | 7.40±0.03 | 0.8778 | 0.8685 |
| Compliance (ml/mbar) | 49±15 | 47±17 | 48±26 | 0.6206 | 0.7824 |
| Infiltrates (quadrants) | 1.07±0.64 | 1.19±0.65 | 1.30±0.63 | 0.0710 | 0.0970 |
| Lung injury score | 1±1 | 1±1 | 1±1 | 0.2189 | 0.2385 |
| ***Scores*** |  |  |  |  |  |
| APACHE II Score | 15.7±7.3 | 14.4±6.7 | 15.1±6.3 | 0.3373 | 0.4931 |
| SAPS II Score | 22.9±5.6 | 23.9±6.8 | 25.2±7.8 | 0.0474 | 0.0976 |
| ***Hemodynamic*** |  |  |  |  |  |
| Heart rate (bpm) | 82±7 | 84±11 | 82±9 | 0.1749 | 0.1328 |
| MAP (mmHg) | 83±7 | 81±7 | 81±7 | 0.4699 | 0.3381 |
| CVP (mmHg) | 11±2 | 11±3 | 11±2 | 0.1627 | 0.1791 |
| PCWP (mmHg), (n) | 12±3 (2) | 15±3 (48) | 15±4 (69) | 0.3153 | 0.3357 |
| PAP sys (mmHg), (n) | 29±0 (2) | 36±9 (56) | 38±8 (77) | 0.1376 | 0.2708 |
| PAP dia (mmHg), (n) | 15±2 (2) | 18±5 (56) | 19±5 (77) | 0.2952 | 0.3216 |
| PAP mean (mmHg), (n) | 26±18 (4) | 26±9 (63) | 26±7 (88) | 0.4120 | 0.9845 |
| CI (l/min/m²), (n) | 3±1 (2) | 3±1 (53) | 3±1 (84) | 0.0322 | 0.2073 |
| SVRI (dyn∙s^-1^∙m²∙cm^-5^), (n) | 1015±0 (1) | 906±225 (48) | 1033±525 (77) | 0.3193 | 0.2843 |
| PVRI (dyn∙s^-1^∙m²∙cm^-5^), (n) | 120±0 (1) | 219±154 (45) | 218±130 (67) | 0.4135 | 0.7810 |
| ***Renal system*** |  |  |  |  |  |
| Urine output (ml/d) | 2112±693 | 1734±714 | 1859±801 | 0.0798 | 0.0534 |
| Urine output (ml/kg/hr) | 1±0 | 1±1 | 1±1 | 0.0401 | 0.1752 |
| Serum creatinine (mg/dl) | 1.2±0.4 | 1.4±1.0 | 1.4±0.6 | 0.2128 | 0.3729 |
| Serum urea (mg/dl) | 21.5±8.3 | 28.1±14.2 | 27.5±13.1 | 0.1496 | 0.1023 |
| Creatinine clearance | 75.2±24.1 | 71.2±31.5 | 68.1±31.5 | 0.1884 | 0.4151 |
| ***Inotropes*** |  |  |  |  |  |
| Epinephrine (mg/d), (n) | 0.1±0.2 (15) | 2.4±14.6 (134) | 1.0±4.8(206) | 0.1731 | 0.2806 |
| Norepinephrine (mg/d), (n) | 0.1±0.1 (10) | 1.4±4.8 (97) | 0.9±3.2 (143) | 0.2494 | 0.2199 |
| Enoximone (mg/d), (n) | 0.2±1.0 (1) | 12.6±69.1 (25) | 6.3±28.9 (37) | 0.4345 | 0.2991 |
| Dobutamine (mg/d), (n) | 1.3±5.5 (2) | 12.2±48.6 (32) | 14.3±47.7 (55) | 0.3565 | 0.4579 |
| ***Other agents*** |  |  |  |  |  |
| NTG (mg/d), (n) | 3.7±5.3 (12) | 5.2±6.8 (129) | 5.2±6.5 (198) | 0.3448 | 0.5784 |
| Amiodarone (mg/d), (n) | 19.7±72.8 (3) | 67.3±212.8 (46) | 74.4±251.0 (79) | 0.2661 | 0.5771 |
| Cortisone (mg/d), (n) | 0.0±0.0 (0) | 34.2±166.1 (22) | 29.1±164.2 (29) | 0.2136 | 0.6536 |
| Vasopressin (iU/d), (n) | 0.0±0.0 (0) | 0.1±0.7 (5) | 0.1±0.8 (7) | 0.7355 | 0.8356 |
| Furosemide (mg/d), (n) | 19.0±17.5 (21) | 36.5±67.1 (158) | 37.4±69.8 (234) | 0.6029 | 0.4817 |
| ***Operative characteristics*** |  |  |  |  |  |
| Cross-clamp time (min) | 99±34 | 95±38 | 94±39 | 0.7564 | 0.8612 |
| Cardiopulmonary bypass time (min) | 147±52 | 145±61 | 145±74 | 0.7354 | 0.9920 |
| Transfused red blood cells (ml/d) | 532±639 | 842±894 | 635±688 | 0.0462 | 0.0136 |
| Fresh frozen plasma (ml/d) | 50±156 | 103±273 | 60±257 | 0.0623 | 0.2267 |
| Prothrombin complex concentrates (iU/d) | 0±0 | 19±126 | 7±85 | 0.3155 | 0.4854 |
| IABP (%) | 0.0 | 5.8 | 6.5 | 0.4786 |  |
| ECMO (%) | 4.8 | 1.0 | 0.0 | 0.0609 |  |
| Length of ICU stay (d) | 6±13 | 7±15 | 7±14 | 0.9112 | 0.9702 |
| Hospitality stay (d) | 21±11 | 25±19 | 25±21 | 0.9684 | 0.7177 |
| ***In-hospital mortality (n=36)*** |  |  |  | 0.3184 |  |
| Overall (7.9%) (%), n=455 | 0.0 | 9.3 | 7.6 |  |  |
| Elective cases (%), n=378 | 0.0 | 7.0 | 5.0 |  |  |
| Urgent cases (%), n=40 | 0.0 | 1.7 | 1.5 |  |  |
| Emergency cases (%), n=37 | 0.0 | 0.6 | 1.2 |  |  |
| Age at surgery (years) | 67±8 | 66±11 | 68±10 | 0.1141 | 0.0680 |
| Survival after surgery (days) | 500±113 | 485±564 | 542±542 | 06600 | 0.8636 |
| Age at death (years) | 71±5 | 72±8 | 74±7 | 0.4925 | 0.4393 |

Table legend: P_a_O_2_/F_i_O_2_: oxygenation index; PEEP: positive end-expiratory pressure; PCO_2_: partial pressure of carbon dioxide; APACHE II Score: Acute Physiology and Chronic Health Evaluation Score; SAPS II Score: Simplified Acute Physiology Score; HR: Heart rate; MAP: mean arterial pressure; CVP: central venous pressure; PCWP: pulmonary capillary wedge pressure; PAP: mean pulmonary artery pressure; CI: cardiac index; SVRI: systemic vascular resistance; PVRI: pulmonary vascular resistance; NTG: nitroglycerin; IABP: intraaortic balloon pump; ECMO: extracorporeal membrane oxygenation; ICU: intensive care unit. The column headings 0, 1 and 2 refer to the frequency of the haplotype; 0 means the haplotype does not exist, 1 means the haplotype appears once and 2 indicates that the haplotype exists twice.

**Peri- and postoperative course associated with *EDN1* H2 (T1370G=G; K198N=T)**

| **Variable** | **0**  (n=316) | **1**  (n=128) | **2**  (n=11) | ***p* value** | ***p* value (F-test)** |
| --- | --- | --- | --- | --- | --- |
| ***Pulmonary function*** |  |  |  |  |  |
| P_a_O_2_/F_i_O_2_ | 260±83 | 249±62 | 271±68 | 0.4529 | 0.3032 |
| PEEP (mbar) | 7±2 | 7±1 | 6±0 | 0.1765 | 0.0881 |
| PCO_2_ (mmHg) | 40±3 | 40±4 | 41±4 | 0.4490 | 0.5448 |
| Arterial pH | 7.40±0.04 | 7.40±0.04 | 7.39±0.03 | 0.3898 | 0.3916 |
| Compliance (ml/mbar) | 47±24 | 47±17 | 51±18 | 0.7013 | 0.8636 |
| Infiltrates (quadrants) | 1.29±0.63 | 1.14±0.68 | 1.15±0.53 | 0.0823 | 0.0842 |
| Lung injury score | 1±1 | 1±1 | 1±0 | 0.5634 | 0.4461 |
| ***Scores*** |  |  |  |  |  |
| APACHE II Score | 14.9±6.4 | 14.7±6.8 | 16.2±7.6 | 0.6649 | 0.7530 |
| SAPS II Score | 25.0±7.9 | 23.7±6.1 | 23.3±5.3 | 0.1149 | 0.1664 |
| ***Hemodynamic*** |  |  |  |  |  |
| Heart rate (bpm) | 81±9 | 85±10 | 81±8 | 0.0011 | 0.0014 |
| MAP (mmHg) | 81±7 | 82±7 | 83±7 | 0.3773 | 0.2456 |
| CVP (mmHg) | 11±2 | 11±3 | 11±2 | 0.3057 | 0.3481 |
| PCWP (mmHg), (n) | 15±4 (80) | 15±3 (38) | 10±0 (1) | 0.2302 | 0.2172 |
| PAP sys (mmHg), (n) | 38±8 (89) | 36±9 (45) | 29±0 (1) | 0.1825 | 0.3701 |
| PAP dia (mmHg), (n) | 19±5 (89) | 18±5 (45) | 16±0 (1) | 0.6776 | 0.7013 |
| PAP mean (mmHg), (n) | 26±8 (104) | 25±9 (49) | 36±23 (2) | 0.3751 | 0.1768 |
| CI (l/min/m²), (n) | 3±1 (95) | 3±1 (43) | 2±0 (1) | 0.0011 | 0.0147 |
| SVRI (dyn∙s^-1^∙m²∙cm^-5^), (n) | 1031±500 (88) | 874±201 (37) | 1015±0 (1) | 0.1296 | 0.1889 |
| PVRI (dyn∙s^-1^∙m²∙cm^-5^), (n) | 224±126 (76) | 208±166 (36) | 120±0 (1) | 0.1093 | 0.6680 |
| ***Renal system*** |  |  |  |  |  |
| Urine output (ml/d) | 1825±781 | 1790±731 | 2172±785 | 0.3579 | 0.2850 |
| Urine output (ml/kg/hr) | 1±1 | 1±1 | 1±0 | 0.2142 | 0.3374 |
| Serum creatinine (mg/dl) | 1.3±0.6 | 1.4±1.1 | 1.3±0.5 | 0.9874 | 0.5350 |
| Serum urea (mg/dl) | 27.1±13.1 | 28.6±14.3 | 23.4±10.2 | 0.5060 | 0.3432 |
| Creatinine clearance | 68.9±31.4 | 71.7±31.2 | 66.9±26.9 | 0.5161 | 0.6593 |
| ***Inotropes*** |  |  |  |  |  |
| Epinephrine (mg/d), (n) | 1.1±5.0 (247) | 2.5±16.6 (100) | 0.1±0.2(8) | 0.4112 | 0.3574 |
| Norepinephrine (mg/d), (n) | 1.0±3.3 (167) | 1.3±5.0 (78) | 0.1±0.1 (5) | 0.1557 | 0.5522 |
| Enoximone (mg/d), (n) | 8.4±45.3 (45) | 9.0±55.7 (18) | 0.0±0.0 (0) | 0.4060 | 0.8371 |
| Dobutamine (mg/d), (n) | 14.0±47.2 (64) | 11.2±48.5 (24) | 0.2±0.8 (1) | 0.5553 | 0.5633 |
| ***Other agents*** |  |  |  |  |  |
| NTG (mg/d), (n) | 5.2±6.6 (238) | 4.9±6.6 (94) | 5.8±6.6 (7) | 0.8168 | 0.8333 |
| Amiodarone (mg/d), (n) | 72.6±239.4 (92) | 66.6±221.8 (35) | 0.4±1.4 (1) | 0.2721 | 0.5910 |
| Cortisone (mg/d), (n) | 33.6±177.1 (35) | 22.6±121.6 (16) | 0.0±0.0 (0) | 0.4639 | 0.6678 |
| Vasopressin (iU/d), (n) | 0.1±0.7 (8) | 0.1±0.8 (4) | 0.0±0.0 (0) | 0.8077 | 0.8880 |
| Furosemide (mg/d), (n) | 37.7±72.0 (282) | 33.5±57.2 (120) | 26.0±20.9 (11) | 0.3449 | 0.7354 |
| ***Operative characteristics*** |  |  |  |  |  |
| Cross-clamp time (min) | 94±39 | 93±37 | 112±33 | 0.1560 | 0.2977 |
| Cardiopulmonary bypass time (min) | 145±73 | 142±57 | 170±46 | 0.1041 | 0.4388 |
| Transfused red blood cells (ml/d) | 658±713 | 844±915 | 591±627 | 0.1931 | 0.0643 |
| Fresh frozen plasma (ml/d) | 67±262 | 99±263 | 47±157 | 0.1384 | 0.4736 |
| Prothrombin complex concentrates (iU/d) | 10±89 | 16±129 | 0±0 | 0.7738 | 0.8076 |
| IABP (%) | 6.0 | 6.3 | 0.0 | 0.6975 |  |
| ECMO (%) | 0.0 | 0.0 | 9.1 | <0.0001 |  |
| Length of ICU stay (d) | 7±14 | 7±16 | 6±14 | 0.6217 | 0.9523 |
| Hospitality stay (d) | 25±21 | 25±19 | 21±11 | 0.7587 | 0.8090 |
| ***In-hospital mortality (n=36)*** |  |  |  | 0.6095 |  |
| Overall (7.9%) (%), n=455 | 8.2 | 7.8 | 0.0 |  |  |
| Elective cases (%), n=378 | 5.4 | 6.3 | 0.0 |  |  |
| Urgent cases (%), n=40 | 1.9 | 0.8 | 0.0 |  |  |
| Emergency cases (%), n=37 | 1.0 | 0.8 | 0.0 |  |  |
| Age at surgery (years) | 68±10 | 66±10 | 66±9 | 0.1280 | 0.2403 |
| Survival after surgery (days) | 529±553 | 495±529 | 576±0 | 0.8932 | 0.9497 |
| Age at death (years) | 74±7 | 72±7 | 67±0 | 0.2141 | 0.3334 |

Table legend: PaO2/FiO2: oxygenation index; PEEP: positive end-expiratory pressure; PCO2: partial pressure of carbon dioxide; APACHE II Score: Acute Physiology and Chronic Health Evaluation Score; SAPS II Score: Simplified Acute Physiology Score; HR: Heart rate; MAP: mean arterial pressure; CVP: central venous pressure; PCWP: pulmonary capillary wedge pressure; PAP: mean pulmonary artery pressure; CI: cardiac index; SVRI: systemic vascular resistance; PVRI: pulmonary vascular resistance; NTG: nitroglycerin; IABP: intraaortic balloon pump; ECMO: extracorporeal membrane oxygenation; ICU: intensive care unit. The column headings 0, 1 and 2 refer to the frequency of the haplotype; 0 means the haplotype does not exist, 1 means the haplotype appears once and 2 indicates that the haplotype exists twice.

**Peri- and postoperative course associated with *EDN1* H3 (T1370G=T; K198N=T)**

| **Variable** | **0**  (n=399) | **1**  (n=56) | **2**  (n=0) | ***p* value** | ***p* value (F-test)** |
| --- | --- | --- | --- | --- | --- |
| ***Pulmonary function*** |  |  |  |  |  |
| P_a_O_2_/F_i_O_2_ | 256±77 | 268±85 |  | 0.2458 | 0.2771 |
| PEEP (mbar) | 7±1 | 7±2 |  | 0.2764 | 0.0786 |
| PCO_2_ (mmHg) | 40±4 | 40±3 |  | 0.3799 | 0.4572 |
| Arterial pH | 7.40±0.04 | 7.39±0.04 |  | 0.7447 | 0.4421 |
| Compliance (ml/mbar) | 48±23 | 45±14 |  | 0.6485 | 0.4885 |
| Infiltrates (quadrants) | 1.25±0.64 | 1.25±0.63 |  | 0.9241 | 0.9569 |
| Lung injury score | 1±1 | 1±1 |  | 0.3336 | 0.7014 |
| ***Scores*** |  |  |  |  |  |
| APACHE II Score | 15.0±6.5 | 13.7±6.5 |  | 0.0912 | 0.1638 |
| SAPS II Score | 24.7±7.3 | 24.0±8.1 |  | 0.3303 | 0.5342 |
| ***Hemodynamic*** |  |  |  |  |  |
| Heart rate (bpm) | 83±10 | 81±9 |  | 0.1351 | 0.1314 |
| MAP (mmHg) | 81±7 | 81±7 |  | 0.5524 | 0.6072 |
| CVP (mmHg) | 11±3 | 11±2 |  | 0.5840 | 0.3898 |
| PCWP (mmHg), (n) | 15±4 (109) | 15±4 (10) |  | 0.9122 | 0.8289 |
| PAP sys (mmHg), (n) | 37±8 (124) | 35±10 (11) |  | 0.5225 | 0.4189 |
| PAP dia (mmHg), (n) | 18±5 (124) | 16±6 (11) |  | 0.2937 | 0.1971 |
| PAP mean (mmHg), (n) | 26±8 (141) | 27±11 (14) |  | 0.7740 | 0.8023 |
| CI (l/min/m²), (n) | 3±1 (129) | 3±0 (10) |  | 0.8898 | 0.7305 |
| SVRI (dyn∙s^-1^∙m²∙cm^-5^), (n) | 986±448 (117) | 973±268 (9) |  | 0.8833 | 0.9320 |
| PVRI (dyn∙s^-1^∙m²∙cm^-5^), (n) | 215±142 (106) | 264±93 (7) |  | 0.0567 | 0.3686 |
| ***Renal system*** |  |  |  |  |  |
| Urine output (ml/d) | 1847±781 | 1657±645 |  | 0.1183 | 0.0842 |
| Urine output (ml/kg/hr) | 1±1 | 1±0 |  | 0.1658 | 0.2073 |
| Serum creatinine (mg/dl) | 1.4±0.8 | 1.2±0.8 |  | 0.0270 | 0.2055 |
| Serum urea (mg/dl) | 27.8±13.4 | 24.8±12.8 |  | 0.0640 | 0.1171 |
| Creatinine clearance | 68.9±31.3 | 74.8±30.4 |  | 0.1385 | 0.1874 |
| ***Inotropes*** |  |  |  |  |  |
| Epinephrine (mg/d), (n) | 1.5±10.2 (312) | 1.6±5.6 (43) |  | 0.1110 | 0.9122 |
| Norepinephrine (mg/d), (n) | 1.0±3.8 (225) | 1.4±3.8 (25) |  | 0.1029 | 0.5377 |
| Enoximone (mg/d), (n) | 7.3±39.5 (56) | 16.4±87.0 (7) |  | 0.7593 | 0.1790 |
| Dobutamine (mg/d), (n) | 13.0±47.4 (80) | 12.1±43.9 (9) |  | 0.5149 | 0.8968 |
| ***Other agents*** |  |  |  |  |  |
| NTG (mg/d), (n) | 5.2±6.6 (299) | 4.9±6.6 (40) |  | 0.4782 | 0.7542 |
| Amiodarone (mg/d), (n) | 70.2±238.7 (117) | 61.7±174.5 (11) |  | 0.1971 | 0.7964 |
| Cortisone (mg/d), (n) | 26.4±149.8 (46) | 52.8±226.5 (5) |  | 0.6665 | 0.2528 |
| Vasopressin (iU/d), (n) | 0.1±0.8 (11) | 0.0±0.2 (1) |  | 0.6643 | 0.4802 |
| Furosemide (mg/d), (n) | 36.2±65.2 (363) | 36.6±81.0 (50) |  | 0.7318 | 0.9650 |
| ***Operative characteristics*** |  |  |  |  |  |
| Cross-clamp time (min) | 95±39 | 92±35 |  | 0.6914 | 0.6158 |
| Cardiopulmonary bypass time (min) | 146±69 | 139±62 |  | 0.6328 | 0.5362 |
| Transfused red blood cells (ml/d) | 704±770 | 742±832 |  | 0.8584 | 0.7298 |
| Fresh frozen plasma (ml/d) | 71±257 | 107±284 |  | 0.2874 | 0.3360 |
| Prothrombin complex concentrates (iU/d) | 11±104 | 10±75 |  | 0.9979 | 0.9498 |
| IABP (%) | 6.3 | 3.6 |  | 0.4242 |  |
| ECMO (%) | 0.0 | 0.0 |  | 0.1039 |  |
| Length of ICU stay (d) | 7±14 | 7±13 |  | 0.2608 | 0.8575 |
| Hospitality stay (d) | 25±20 | 24±19 |  | 0.5124 | 0.7619 |
| ***In-hospital mortality (n=36)*** |  |  |  | 0.4067 |  |
| Overall (7.9%) (%), n=455 | 7.5 | 10.7 |  |  |  |
| Elective cases (%), n=378 | 5.3 | 7.1 |  |  |  |
| Urgent cases (%), n=40 | 1.3 | 3.6 |  |  |  |
| Emergency cases (%), n=37 | 1.0 | 0.0 |  |  |  |
| Age at surgery (years) | 68±10 | 66±12 |  | 0.5525 | 0.1998 |
| Survival after surgery (days) | 539±546 | 369±508 |  | 0.2444 | 0.2881 |
| Age at death (years) | 73±7 | 72±10 |  | 0.9965 | 0.6208 |

Table legend: P_a_O_2_/F_i_O_2_: oxygenation index; PEEP: positive end-expiratory pressure; PCO_2_: partial pressure of carbon dioxide; APACHE II Score: Acute Physiology and Chronic Health Evaluation Score; SAPS II Score: Simplified Acute Physiology Score; HR: Heart rate; MAP: mean arterial pressure; CVP: central venous pressure; PCWP: pulmonary capillary wedge pressure; PAP: mean pulmonary artery pressure; CI: cardiac index; SVRI: systemic vascular resistance; PVRI: pulmonary vascular resistance; NTG: nitroglycerin; IABP: intraaortic balloon pump; ECMO: extracorporeal membrane oxygenation; ICU: intensive care unit. The column headings 0, 1 and 2 refer to the frequency of the haplotype; 0 means the haplotype does not exist, 1 means the haplotype appears once and 2 indicates that the haplotype exists twice.

**Peri- and postoperative course associated with *EDN1* H4 (T1370G=G; K198N=G)**

| **Variable** | **0**  (n=447) | **1**  (n=8) | **2**  (n=0) | ***p* value** | ***p* value (F-test)** |
| --- | --- | --- | --- | --- | --- |
| ***Pulmonary function*** |  |  |  |  |  |
| P_a_O_2_/F_i_O_2_ | 258±78 | 224±76 |  | 0.2909 | 0.2263 |
| PEEP (mbar) | 7±1 | 6±2 |  | 0.1346 | 0.3703 |
| PCO_2_ (mmHg) | 40±3 | 39±3 |  | 0.3644 | 0.4972 |
| Arterial pH | 7.40±0.04 | 7.40±0.03 |  | 0.8974 | 0.8764 |
| Compliance (ml/mbar) | 47±22 | 49±16 |  | 0.5279 | 0.8607 |
| Infiltrates (quadrants) | 1.25±0.64 | 0.94±0.63 |  | 0.1537 | 0.1715 |
| Lung injury score | 1±1 | 1±1 |  | 0.4202 | 0.3077 |
| ***Scores*** |  |  |  |  |  |
| APACHE II Score | 14.8±6.5 | 16.9±7.8 |  | 0.4616 | 0.3720 |
| SAPS II Score | 24.6±7.4 | 22.9±6.2 |  | 0.7341 | 0.5029 |
| ***Hemodynamic*** |  |  |  |  |  |
| Heart rate (bpm) | 83±10 | 77±2 |  | 0.0879 | 0.1409 |
| MAP (mmHg) | 81±7 | 82±6 |  | 0.7243 | 0.6922 |
| CVP (mmHg) | 11±2 | 13±3 |  | 0.0456 | 0.0243 |
| PCWP (mmHg), (n) | 15±3 (117) | 16±7 (2) |  | 0.8361 | 0.6441 |
| PAP sys (mmHg), (n) | 37±8 (133) | 44±5 (2) |  | 0.1897 | 0.2341 |
| PAP dia (mmHg), (n) | 18±5 (133) | 19±7 (2) |  | 0.9637 | 0.9096 |
| PAP mean (mmHg), (n) | 26±9 (151) | 26±11 (4) |  | 0.7264 | 0.9115 |
| CI (l/min/m²), (n) | 3±1 (137) | 2±1 (2) |  | 0.0620 | 0.0627 |
| SVRI (dyn∙s^-1^∙m²∙cm^-5^), (n) | 981±438 (124) | 1186±338 (2) |  | 0.1780 | 0.5126 |
| PVRI (dyn∙s^-1^∙m²∙cm^-5^), (n) | 217±140 (111) | 264±66 (2) |  | 0.2145 | 0.6401 |
| ***Renal system*** |  |  |  |  |  |
| Urine output (ml/d) | 1817±769 | 2158±629 |  | 0.1910 | 0.2138 |
| Urine output (ml/kg/hr) | 1±1 | 1±0 |  | 0.1363 | 0.2670 |
| Serum creatinine (mg/dl) | 1.4±0.8 | 1.0±0.1 |  | 0.1616 | 0.2117 |
| Serum urea (mg/dl) | 27.5±13.5 | 21.1±6.5 |  | 0.2557 | 0.1801 |
| Creatinine clearance | 69.6±31.4 | 72.1±23.1 |  | 0.6215 | 0.8182 |
| ***Inotropes*** |  |  |  |  |  |
| Epinephrine (mg/d), (n) | 1.5±9.8 (350) | 0.2±0.4 (5) |  | 0.5031 | 0.7082 |
| Norepinephrine (mg/d), (n) | 1.1±3.9 (246) | 0.2±0.3 (4) |  | 0.7781 | 0.5142 |
| Enoximone (mg/d), (n) | 8.3±48.1 (61) | 12.5±33.1 (2) |  | 0.3497 | 0.8055 |
| Dobutamine (mg/d), (n) | 13.1±47.4 (88) | 3.1±8.8 (1) |  | 0.5901 | 0.5529 |
| ***Other agents*** |  |  |  |  |  |
| NTG (mg/d), (n) | 5.2±6.6 (334) | 3.3±5.1 (5) |  | 0.3356 | 0.4282 |
| Amiodarone (mg/d), (n) | 69.5±233.5 (124) | 52.0±58.6 (4) |  | 0.1794 | 0.8325 |
| Cortisone (mg/d), (n) | 30.1±162.5 (50) | 5.7±16.1 (1) |  | 0.9309 | 0.6713 |
| Vasopressin (iU/d), (n) | 0.1±0.7 (12) | 0.0±0.0 (0) |  | 0.6390 | 0.7254 |
| Furosemide (mg/d), (n) | 36.5±67.8 (405) | 21.7±26.5 (8) |  | 0.9827 | 0.5379 |
| ***Operative characteristics*** |  |  |  |  |  |
| Cross-clamp time (min) | 94±38 | 112±54 |  | 0.4647 | 0.1910 |
| Cardiopulmonary bypass time (min) | 144±68 | 165±85 |  | 0.6530 | 0.3951 |
| Transfused red blood cells (ml/d) | 711±780 | 585±586 |  | 0.7655 | 0.6504 |
| Fresh frozen plasma (ml/d) | 77±262 | 0±0 |  | 0.3324 | 0.4085 |
| Prothrombin complex concentrates (iU/d) | 10±98 | 74±197 |  | 0.0171 | 0.0944 |
| IABP (%) | 6.0 | 0.0 |  | 0.4735 |  |
| ECMO (%) | 0.5 | 0.0 |  | 0.8496 |  |
| Length of ICU stay (d) | 7±14 | 3±2 |  | 0.7137 | 0.4361 |
| Hospitality stay (d) | 25±20 | 16±2 |  | 0.3818 | 0.2383 |
| ***In-hospital mortality (n=36)*** |  |  |  | 0.4029 |  |
| Overall (7.9%) (%), n=455 | 8.1 | 0.0 |  |  |  |
| Elective cases (%), n=378 | 5.6 | 0.0 |  |  |  |
| Urgent cases (%), n=40 | 1.6 | 0.0 |  |  |  |
| Emergency cases (%), n=37 | 0.9 | 0.0 |  |  |  |
| Age at surgery (years) | 67±10 | 70±8 |  | 0.3448 | 0.3961 |
| Survival after surgery (days) | 511±535 | 1580±0 |  | 0.1169 | 0.0490 |
| Age at death (years) | 73±7 | 84±0 |  | 0.0999 | 0.1297 |

Table legend: P_a_O_2_/F_i_O_2_: oxygenation index; PEEP: positive end-expiratory pressure; PCO_2_: partial pressure of carbon dioxide; APACHE II Score: Acute Physiology and Chronic Health Evaluation Score; SAPS II Score: Simplified Acute Physiology Score; HR: Heart rate; MAP: mean arterial pressure; CVP: central venous pressure; PCWP: pulmonary capillary wedge pressure; PAP: mean pulmonary artery pressure; CI: cardiac index; SVRI: systemic vascular resistance; PVRI: pulmonary vascular resistance; NTG: nitroglycerin; IABP: intraaortic balloon pump; ECMO: extracorporeal membrane oxygenation; ICU: intensive care unit. The column headings 0, 1 and 2 refer to the frequency of the haplotype; 0 means the haplotype does not exist, 1 means the haplotype appears once and 2 indicates that the haplotype exists twice.
